# Blood-Enriching Effects and Immune-Regulation Mechanism of Steam-Processed *Polygonatum Sibiricum* Polysaccharide in Blood Deficiency Syndrome Mice

**DOI:** 10.3389/fimmu.2022.813676

**Published:** 2022-02-17

**Authors:** Juan Wang, Furong Wang, Lixia Yuan, Hongsheng Ruan, Zhibiao Zhu, Xiaoling Fan, Lingyan Zhu, Xin Peng

**Affiliations:** ^1^ Department of Traditional Chinese Medicine, Zhejiang Pharmaceutical College, Ningbo, China; ^2^ Department of Quality Control, Zhejiang Sanxitang Chinese Medicine Co., LTD, Yiwu, China; ^3^ Ningbo Research Institute of Zhejiang University, Ningbo, China

**Keywords:** steam-processed *Polygonatum sibiricum* polysaccharide, blood deficiency syndrome, RNA-seq, JAK1-STAT1, hematopoietic cytokines

## Abstract

*Polygonatum sibiricum* Red. has been used as a medicinal herb and nutritional food in traditional Chinese medicine for a long time. It must be processed prior to clinical use for safe and effective applications. However, the present studies mainly focused on crude *Polygonatum sibiricum* (PS). This study aimed to investigate the chemical properties, blood-enriching effects and mechanism of polysaccharide from the steam-processed *Polygonatum sibiricum* (SPS), which is a common form of PS in clinical applications. Instrumentation analyses and chemistry analyses revealed the structure of SPS polysaccharide (SPSP). A mice model of blood deficiency syndrome (BDS) was induced by acetylphenylhydrazine (APH) and cyclophosphamide (CTX). Blood routine test, spleen histopathological changes, serum cytokines, etc. were measured. The spleen transcriptome changes of BDS mice were detected by RNA sequencing (RNA-seq). The results showed that SPSP consists predominantly of Gal and GalA together with fewer amounts of Man, Glc, Ara, Rha and GlcN. It could significantly increase peripheral blood cells, restore the splenic trabecular structure, and reverse hematopoietic cytokines to normal levels. RNA-seq analysis showed that 122 differentially expressed genes (DEGs) were obtained after SPSP treatment. GO and KEGG analysis revealed that SPSP-regulated DEGs were mainly involved in hematopoiesis, immune regulation signaling pathways. The reliability of transcriptome profiling was validated by quantitative real-time PCR and Western blot, and the results indicated that the potential molecular mechanisms of the blood-enriching effects of SPSP might be associated with the regulating of JAK1-STAT1 pathway, and elevated the hematopoietic cytokines (EPO, G-CSF, TNF-α and IL-6). This work provides important information on the potential mechanisms of SPSP against BDS.

## Introduction

In Traditional Chinese Medicine (TCM), blood deficiency is considered to be related to deficiency in the stomach and spleen, and insufficient hematogenesis, which resembles to the symptoms of anemia in modern medicine (1). TCM considers Qi and blood as the primary factors of blood deficiency, and blood deficiency syndrome (BDS) is a pathological state caused by weakness of spleen, excessive blood loss, andaplastic anemia, and other blood diseases (2). In clinal, patients such as postoperative and postpartum women with chronic bleeding, and women with excessive menstruation usually have symptoms similar to BDS (2). It is usually diagnosed by lower amounts of hemoglobin, blood cells and so on (3). Hematopoietic cytokines, such as thrombopoietin (TPO), erythropoietin (EPO), interleukin-6 (IL-6), and granulocyte colony stimulating factor (G-CSF), play vital roles in the progress of haemopoiesis (4).

Oral iron supplements are the most conventional and effective agents to cure BDS. However, its application is limited for the adverse effects, such as gastrointestinal discomfort and exacerbate inflammatory bowel disease in clinic (5). In recent years, several Chinese medicinal herb recipes that can enrich and regulate blood, such as E’jiao, *Panax notoginseng*, *Angelica sinensis*, Siwu Decoction have been proved that they have promising effects on prevention and treatment of BDS as well as anemia (6–9).

The dry rhizome of *Polygonatum sibiricum* Red. is called as *Polygonatum sibiricum* (PS). It possesses a strong effect on invigorating Qi, nourishing Yin, moistening the lung, and tonifying spleen and kidney. Besides, PS can be used to treat coughing, weakness, fatigue, indigestion, premature graying of hair, anti-aging, etc. (10). Since its beneficial effects on prolonging one’s life, PS is considered as the “Top grade” herbs in *Shennong Bencao Jing*, one of the most respected Chinese classics about medicinal plants. Numbers of active compounds have been isolated from PS, mainly including polysaccharides, monosaccharides, saponins and many other bioactive substances (11). Specifically, polysaccharides of PS exhibited many biological activities such as anti-tumor (12), enhancing immunity (13, 14), antioxidant (13, 15), regulating blood glucose and lipids (13, 16), lung protection activities (17), etc. The mixture of fructose, glucose, and sucrose in PS was reported to have blood tonic activity (18).

For safe and effective applications, PS must be processed prior to clinical use, which usually processed by steam or rice wine to eliminate the side effects of numbing (19). A study had reported that the types and molar ratios of monosaccharides were different between wine-processed PS polysaccharides (WPSP) and crude PS polysaccharides (CPSP), and the immunological activity *in vitro* and vivo of WPSP was significantly better than that of CPSP (20). Clinical studies have demonstrated that steam-processed PS (SPS) could enhance the original function of “invigorating qi and blood, and tonifying the spleen, kidney and liver” (21). An animal study revealed that SPS could significantly increase WBC, RBC, HGB, and PLT in peripheral blood of BDS mice, while there were not significantly improved in crude PS (22). Nevertheless, little is known regarding the chemical properties of SPS polysaccharides (SPSP), and the potential molecular mechanism on BDS. Therefore, the chemical characteristics of SPSP and its mechanism on blood deficiency are very worthy of further research. In this study, instrumentation analyses and chemistry analyses were utilized together to identify the structure of SPSP. Cyclophosphamide (CTX) and acetyl phenylhydrazine (APH) were used to establish the haemolytic, aplastic anaemia and BDS model (23, 24). The effects of SPSP on BDS were evaluated with a classical BDS model induced by CTX and APH in this study. Transcriptomics was used to explore the potential mechanism of SPSP on BDS mice.

## Materials and Methods

### Materials and Chemicals

The steam-processed *Polygonatum sibiricum* was provided by Zhejiang Sanxitang Traditional Chinese Medicine Co., Ltd (No. Y2012301, Wuyi, China). Voucher specimens were deposited at Zhejiang Pharmaceutical College, Ningbo, China. CTX was obtained from Hengrui Medicine Co., Ltd. (Jiangsu, China). APH was purchased from Aladdin Biochemical Technology Co., Ltd (Shanghai, China). ELISA detection kits for G-CSF, tumor necrosis factor-*α* (TNF-*α*), IL-6, and EPO were purchased from Shanghai Jianglai Biological Technology Co., Ltd. (Shanghai, China).

### Extraction, Isolation, and Purification of SPSP

1.2 kg steam-processed *Polygonatum sibiricum* were soaked in deionized water at a ratio of 1:10 (w/v) for 12 h and refluxed twice at 80°C, 1.5 h each. The extracts were combined together and concentrated to a suspension of a solid–liquid ratio of 1:1 by a rotary evaporator at 60°C. Anhydrous ethanol was slowly added to the suspension to make its final concentration be 80%. Then, the suspension was stored at 4°C overnight to obtain the precipitates. The precipitates were collected by centrifugation and heated on a 60°C water bath to remove the residual ethanol. Subsequently, the precipitates were dissolved in an appropriate amount of ultrapure water, and deproteinized using Sevag reagent (n-butanol:chloroform = 1:4, v/v). The deproteinized solution was dialyzed with running distilled water (cut-off Mw: 8000 Da) for 72 h. Following that, the deproteinized samples were observed on a UV spectrophotometer, which revealed no absorbance peaks at 280 nm. Finally, a SPSP sample was obtained through vacuum freeze drying for subsequent experiments.

### Partial Structural Characterization of SPSP

#### Molecular Weights (Mw)

The Mw of SPSP was measured using high performance gel permeation chromatography (HPGPC) system following a previously reported method (25). SPSP was dissolved in 0.02 M phosphate buffer (pH 6.8) at a concentration of 5 mg/mL. Dextran standards with different Mw (1152, 11600, 23800, 48600, 80900, 148000, 273000, and 409800 Da) were used to obtain the standard calibration curve. Both the sample and standard solutions were centrifugated at 13000 r·•min^-1^ for 10 min, and 20 *μ*L of supernatant were injected into a HPGPC instrument (Column: Shodex SB-804, 300×8 mm), respectively. The column temperature was kept constant at 25°C, and the mobile phase was phosphate buffer at a flow rate of 0.5 mL•min^-1^.

#### Monosaccharide Composition Analysis

3 M trifluoroacetic acid (TFA) solution was used to dissolve SPSP (10 mg), and then the solute (1 mg/mL) was hydrolyzed at 120°C for 3 h. A rotary evaporator was used to evaporate the hydrolysate before mixing with methanol, followed by another evaporation session to complete dryness with a termovap sample concentrator. Then the residue was dissolved in distilled water (5 mL), and diluted to 50 mL. The Ion chromatography (IC) measurements was performed with a Dionex ICS5000 system (ThermoFisher, USA) equipped with an anion exchange column (Dionex CarboPac PA20, 3 mm×150 mm). The column temperature was kept constant at 30°C. Sixteen standard monosaccharides, namely fucose (Fuc), rhamnose (Rha), arabinose (Ara), N-acetyl-D-glucosamine (GlcNAc), galactose (GalA), mannose (Man), glucose (Glc), xylose (Xyl), mannuronic acid (ManA), fructose (Fru), ribose (Rib), Galactosamine hydrochloride (GalN), Glucosamine hydrochloride (GlcN), guluronic acid (GulA), glucuronic acid (GlcA), and galacturonic acid (GalA) were used as the references.

#### Fourier Transform-Infrared (FT-IR) Spectra Analysis

2 mg dry SPSP powder were blended along with KBr, and pressed into sheets in a mold. A Perkin-Elmer FT-IR spectrometer from 4000 to 400 cm^−1^ was used to collect the samples’ FT-IR spectra (26).

#### Conformational and Morphological Analysis

The surface morphology of SPSP was observed by a scanning electron microscope (Nova Nano SEM 230, Fei Czech Republic S.r.o). The dried SPSP was scattered on a specimen holder with double-sided tape and covered with a thin gold coating. The sample was observed with 5000-fold and 50000-fold magnifications under a low vacuum condition.

#### X-Ray Diffraction (XRD) Analysis

A drop of SPSP solution was loaded on a glass slide and measured with an X-ray diffractometer (XRD-DY5261/Xpert3, CEM, USA) with Cu kα radiation (40 kV and 40 mA). Diffractograms were taken at the 2θ angles of 5° to 90°.

### Experimental Design *In Vivo*

#### Blood Deficiency Syndrome (BDS) Model and Treatments

Male and female (1:1) Balb/c mice (18–22 g) were purchased from Zhejiang Provincial Academy of Medical Sciences [(Certificate No. SCXK (Zhe)20190002)]. All animals were fed under standard conditions (24 ± 2°C, 50 ± 2% RH, and 12-12 h light-dark cycle) with food and water fed ad libitum.

After acclimatization for one week, 50 mice were randomly divided into five groups (10 mice each), namely the control group, BDS model group, the Dangguibuxue oral liquid (DOL) group, the high-dose SPSP (H-SPSP) group, and the low-dose SPSP (L-SPSP) group. Distilled water was administered daily to the mice in the control and model groups intragastrically once daily, and the other groups received DOL (3 mL/kg), H-SPSP (400 mg/kg), and L-SPSP (100 mg/kg) treatments daily for 14 consecutive days, respectively. Meanwhile, the BDS model was established by hypodermic injecting APH saline solution hypodermically on days 2 and 5 at doses of 20 and 40 mg/kg, respectively. Two hours after the second injection of APH saline solution, CTX saline solution was injected into the mice intraperitoneally for 4 consecutive days at a dose of 40 mg/kg to reproduce the BDS model (3). In the meantime, mice in the control group were injected with 0.9% normal saline in an equivalent amount following the same procedure.

#### Blood Routine Test

On day 14, mice were anesthetized with sodium pentobarbital, and orbit blood samples were collected into EDTA•K_2_ anticoagulant tubes and analyzed a XT1800i full-automatic blood cell analyzer (SYSMEX, Kobe, Japan) to quantify hemoglobin (HGB), red blood cell (RBC), white blood cell (WBC), hematocrit (HCT), red cell volume distribution width (RDW), and packed cell volume (PCV).

#### Body Temperature (BT), Body Weight Gain (BWG) and Spleen Index (SI)

The BT of mice was measured by an electronic thermometer on day 14. The BW of each group were weighed on day 1 (BW1) and day 14 (BW14). The spleen were removed and weighed after blood collection. The body weight gain (BWG) value was calculated as BWG(%)=(BW14-BW1)/BW1*100%, and the spleen index (mg/g) = (spleen weight/body weight).

#### Spleen Histopathological Changes

Spleen samples were fixed in 10% neutral buffered formalin, and then embedded in paraffin. Sample sections with a thickness of 6 µm were cut from the paraffin blocks by a rotary microtome (Leica Biosystems, Wetzlar, Germany) and stained with hematoxylin-eosin (HE) to observe the pathological change in the spleen. Analysis were performed under a light microscope and photographed *via* ×20 objective lenses.

#### Analysis of EPO, G-CSF, TNF-α and IL-6 in Mice Serum

Blood samples from the orbital vein were centrifuged at 3000 r•min^-1^ (10 min, 4 °C) to obtain the supernatant. The activities of hematopoietic function factors (EPO, G-CSF), and immunomodulatory factors (TNF-*α*, IL-6) in serum were determined with enzyme-linked immunoassays. Operations were conducted following the manufacturer’s instructions.

### RNA Sequencing (RNA-Seq)

The RNA extraction, cDNA library construction and RNA-seq were completed at Biotranstech (Shanghai, China). Total RNA was isolated from spleen tissue using Trizol reagent (Invitrogen, USA). RNA quality was assessed by a 2100 Bioanalyzer (Agilent, USA), and quantification was determined with a ND-2000 spectrophotometer (Thermo Fisher Scientific Inc., USA). Library construction was performed using TruSeq protrol (Illumina, USA), and followed by sequencing on Illumina Novaseq platform. The RNA-Seq was sequenced with 2×150 bp pair-ed strategy. The control group, model group and SPSP group was comprised of three biological and technical replicates. The clean data were obtained by discarding low-quality RNA-Seq reads (Qscore < 30).

### RNA-Seq Data Analysis

The differentially expressed genes (DEGs) analysis was completed using the EdgeR package in R (https://r-project.org) (27). DEGs were screened out under the conditions (Fold Change ≥ 2 and Q-values ≤ 0.005). Venny 2.1.0 was conducted for the common and unique DEGs between the groups. Hierarchical clustering was used to compare the expression of DEGs in model vs control and SPSP vs model groups using cluster analysis software. Gene ontology (GO), pathway annotation, and enrichment analyses were carried out using Goatools (https://github.com/tanghaibao/GOatools) and KOBAS(http://kobas.cbi.pku.edu.cn/kobas3/?t=1). String11.0 was used to obtain the protein-protein interaction (PPI) network and Cytoscape 3.8.2 software was used to make it visualized.

### Real-Time Quantitative PCR (RT-qPCR)

According to the manufacturer’s protocols, frozen spleen tissues (100 mg) were mixed with 1 mL Trizol reagents (Invitrogen) to extract total RNA. Equal amounts of total RNA was reverse-transcribed into cDNA using the PrimeScript RT reagent kit with gDNA Eraser (Takara, Japan). Primer sequences (5′-3′) used in the experiment were shown as follows: JAK1 (250bp) forward: TGGAGGTAACCACATAGC, reverse: CCGAGAACCCAAATAGTC. STAT1 (107bp) forward: AAACTGCCAACTCAACACCTCT, reverse: AGACCACCTCTCTTCCTGTCGT. EPO (90 bp): forward: CGCTTGGAAGACTTGGTGTGT, reverse: CTCACCCTCGAGCTGGTATGA. GATA1 (110 bp): forward: GGAGGGACAGGACAGGTCACT, reverse: GTTTGCTGACAATCATTCGCTT. GAPDH (95 bp) forward: AGGTCGGTGTGAACGGATTTG, reverse: GGGGTCGTTGATGGCAACA. RT-qPCR was performed in 20 μL reactions to detect the expressions of Jak1 and Stat1 mRNAs in spleen tissues using a Light Cycler 96 (Roche, Germany). Amplification parameters included an initial phase of 95°C for 2 min followed by 40 cycles of 60°C for 34 s and 72°C for 30 s.

### Western Blotting

Total proteins were extracted from spleen tissues with a extraction Kit (KeyGen BioTECH, Nanjing, China), and the protein samples (30 *μ*g) was separated onto a 8% SDS-PAGE gel (STAT1), 10% SDS-PAGE gel (JAK1, GATA1, EPOR), and then transferred onto polyvinylidene difluoride (PVDF) membranes. The membranes were blocked with 5% non-fat milk for 2 h, and then washed with Tris buffered saline Tween (TBST) for three times, 5 minutes each. Thereafter, PVDF membranes were incubated with primary antibodies anti-JAK1(1:1000), (Abcam, Cambridge, USA), anti-STAT1(1:500), anti-GATA1(1:500), anti-EPOR (1:500), and anti-GAPDH (1:1000), (Affinity, California, USA), at 4°C overnight. All membranes were washed with TBST buffer for 3 times, 10 minutes each, and then incubated with secondary antibodies at room temperature for 2 h, respectively. All immunoreactive bands were visualized with a ChemiDoc MP Imaging System (Bio-rad, USA) and analyzed by Gel-Pro32 software.

### Statistical Analysis

The results are expressed as the means ± standard deviation (SD). SPSS 22.0 was employed for data analysis. Statistical significance was determined by one-way analysis of variances (ANOVA), and *p*<0.05 was considered statistically significant.

## Results

### Molecular Weights

**Figure 1:**
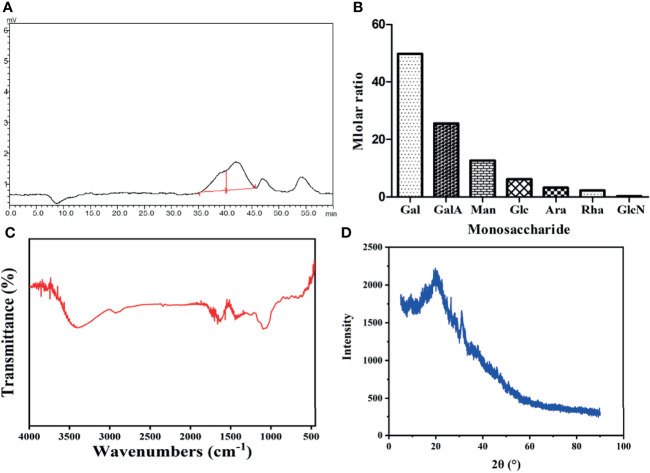
A combination of instrumentation analysis and chemistry analyses of SPSP. **(A)** HPGPC analysis of SPSP. **(B)** monosaccharide composition of SPSP. **(C)** FT-IR spectrum. **(D)** XRD spectroscopy of SPSP film.

The molecular weight of a polysaccharide is closely related to its bioactivity (28). As shown in **Figure 1A**, SPSP exhibited two main peaks with molecular weights of 3094 Da and 14846 Da, respectively.

### Monosaccharide Composition Analysis

As shown in **Figure 1B** and **Figure S1**, ICS analysis showed that the major monosaccharide components of SPSP were Gal and GalA, followed by lower amounts of Man, Glc, Ara, Rha and GlcN in molar ratios of 49.7: 25.6: 12.7:6.2:3.2:2.3:0.3.

### FT-IR Spectra Analysis

The FT-IR spectrum of SPSP was shown in **Figure 1C**. The strong and broad absorption bands at 3452 cm^-1^ were attributed to the stretching vibration of O-H (14). The weak band around 2935 cm^-1^ was -CH_2_- (29). The absorbance around 1700 cm^−1^ was presumed to be C=O groups, suggesting the presence of uronic acid in SPSP. The absorption peak at 1435 cm^-1^ was the stretching vibration of carboxylate anions (COO^-^), demonstrating the existence of free carboxyl groups (25). The stretching peak around 1108 cm^-1^ reflected the presence of C-O-C glycosidic bonds and pyranoid rings (29). The absorption peak around 800 cm^-1^ was characteristic of *α*-type glycosidic bonds (30). These results suggested that SPSP had the characteristic absorption spectrum of common polysaccharides.

### XRD Analysis

The XRD pattern of SPSP was depicted in **Figure 1D**. It could be seen from the map that the characteristic absorption peak intensity of SPSP became weaker and gradually disappeared, suggesting weak crystalline properties of SPSP. Broad peaks around 2θ = 20° could be observed, indicating SPSP was not a polymer with the coexistence of sub-crystallines and amorphous crystals.

### SEM Analysis

**Figure 2:**
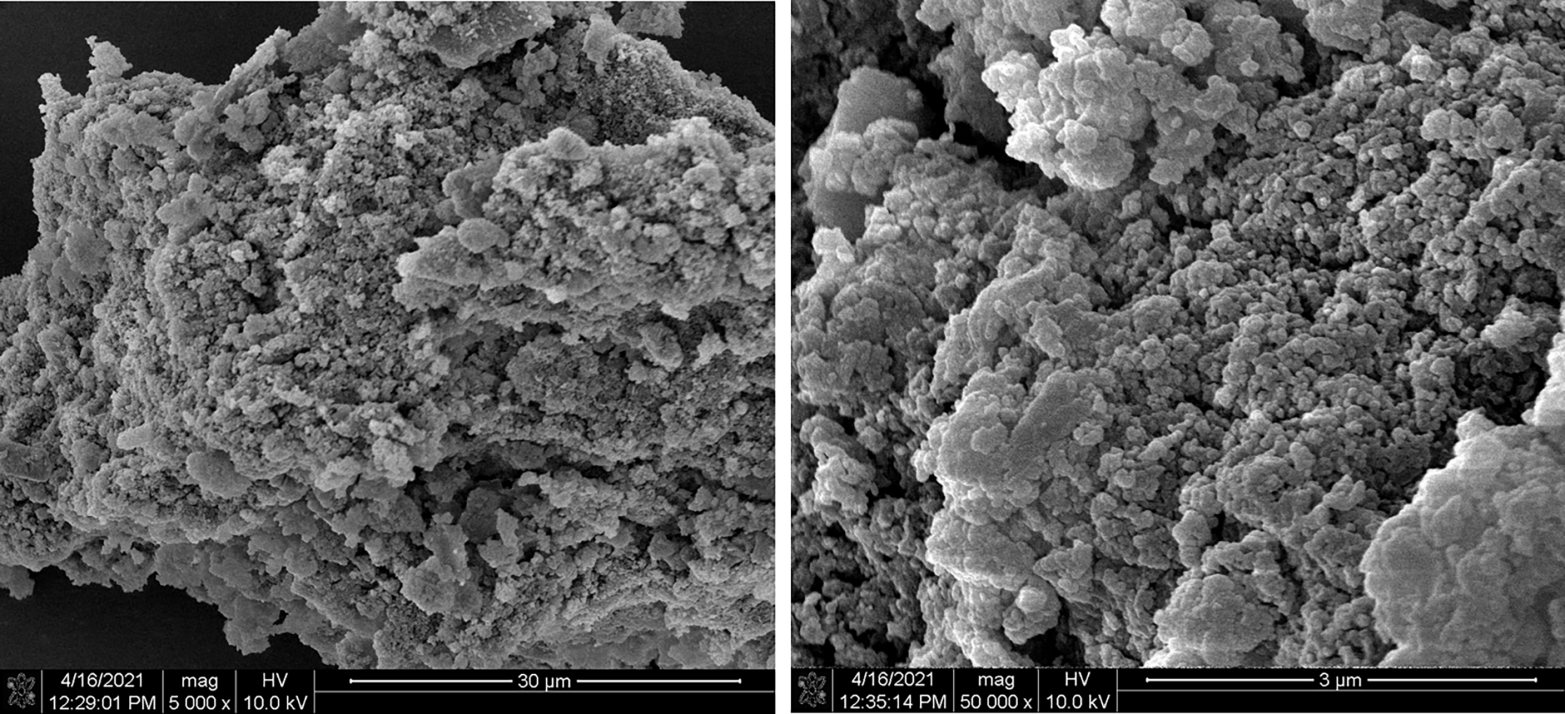
SEM analysis of SPSP.

The morphological analysis of SPSP was reflected clearly in the SEM images. As shown in **Figure 2**, in the scale bar of 300 *μ*m, SPSP had an uneven, rough surface with irregularly particulate matters. Such irregularity may be the result of phase separation of the essential oil, which coalesced from the SPS extract and stayed in the polymer matrix after solvent evaporation (31). Furthermore, in the scale bar of 3 *μ*m, SPSP presented a reticular structure with compact and porous configurations in the middle, ensuring good hydrophilicity of SPSP.

### BT, BWG and Spleen Index (SI)

As shown in **Table 1**, compared to the control group, the ratios of BWG and BT in the BDS group decreased notably (*p*<0.01), while the spleen index elevated abnormally (*p*<0.01). Compared to the BDS model group, the BT of the L-SPSP, H-SPSP and DOL groups showed significant increases (*p*<0.01), while the SI of L-SPSP and H-SPSP groups decreased remarkably (*p*<0.01). Finally, the BWG ratio demonstrated no significant differences between BDS model group and SPSP groups (*p*>0.05).

**Table 1:** Effect of SPSP on ratio of BWG, BT and SI.

Groups	Dose (g/kg)	Ratio of BWG (%)	BT (°C)	SI (%)
Control	–	31.60 ± 9.09	37.92 ± 0.27	0.89 ± 0.10
BDS	–	18.61 ± 11.39^##^	36.54 ± 0.59^##^	1.64 ± 0.37^##^
L-SPSP	0.4	19.90 ± 11.05	37.47 ± 0.62**	1.16 ± 0.38**
H-SPSP	0.1	23.39 ± 11.76	37.59 ± 0.52**	1.13 ± 0.23**
DOL	3 ml/kg	15.77 ± 9.17	37.51 ± 0.56**	1.49 ± 0.26

Values given are the means ± SD, with n =10. ^##^p < 0.01 versus Control group. ^**^p < 0.01 versus BDS. Statistical significant differences were determined using a one-way ANOVA followed by Dunnett’s multiple comparisons test or post hoc analysis.

### Blood Routine Test

**Figure 3:**
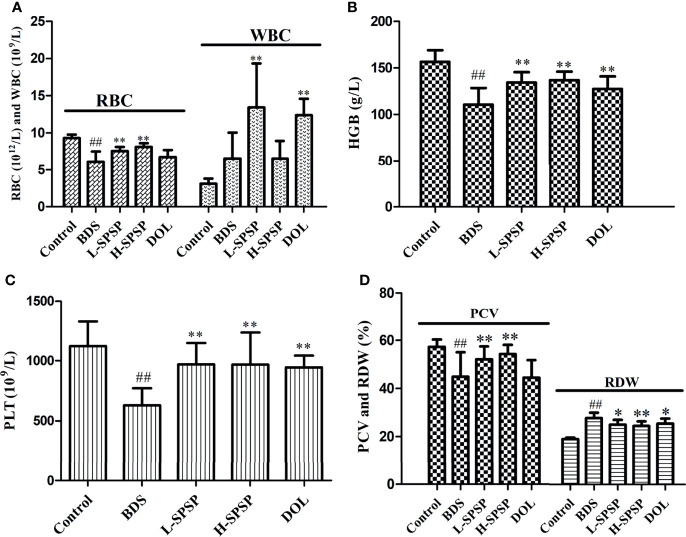
Blood parameters of the BDS mice after treatment with different doses of SPSP. **(A)** RBC and WBC numbers. **(B)** The content of HGB. **(C)** The number of PLT. **(D)** The ratio of PCV and RDW. Each value is represented as the mean ± SD (n = 8); ^##^ *p* < 0.01 versus Control group. ^*^ *p* < 0.05, ^**^ *p* < 0.01 versus BDS.

Fourteen days after administering SPSP or DOL, the quantities of WBC, RBC, HGB, PLT, PCV and RDW in the mice peripheral blood were measured (**Figure 3**). As compared to the control group, the contents of RBC, HGB, PLT, and PCV in the BDS group dropped significantly (*p*<0.01), while the levels of WBC and RDW rose significantly, suggesting the successful establishment of the anemia model. Compared with the BDS group, levels of WBC, RBC, HGB, PLT and PCV in mice treated with SPSP or DOL rose significantly (*p*< 0.01 or *p*< 0.05), but the RDW levels reduced significantly (*p*< 0.01 or *p*< 0.05).

### Histopathological Features of the Spleen

**Figure 4:**
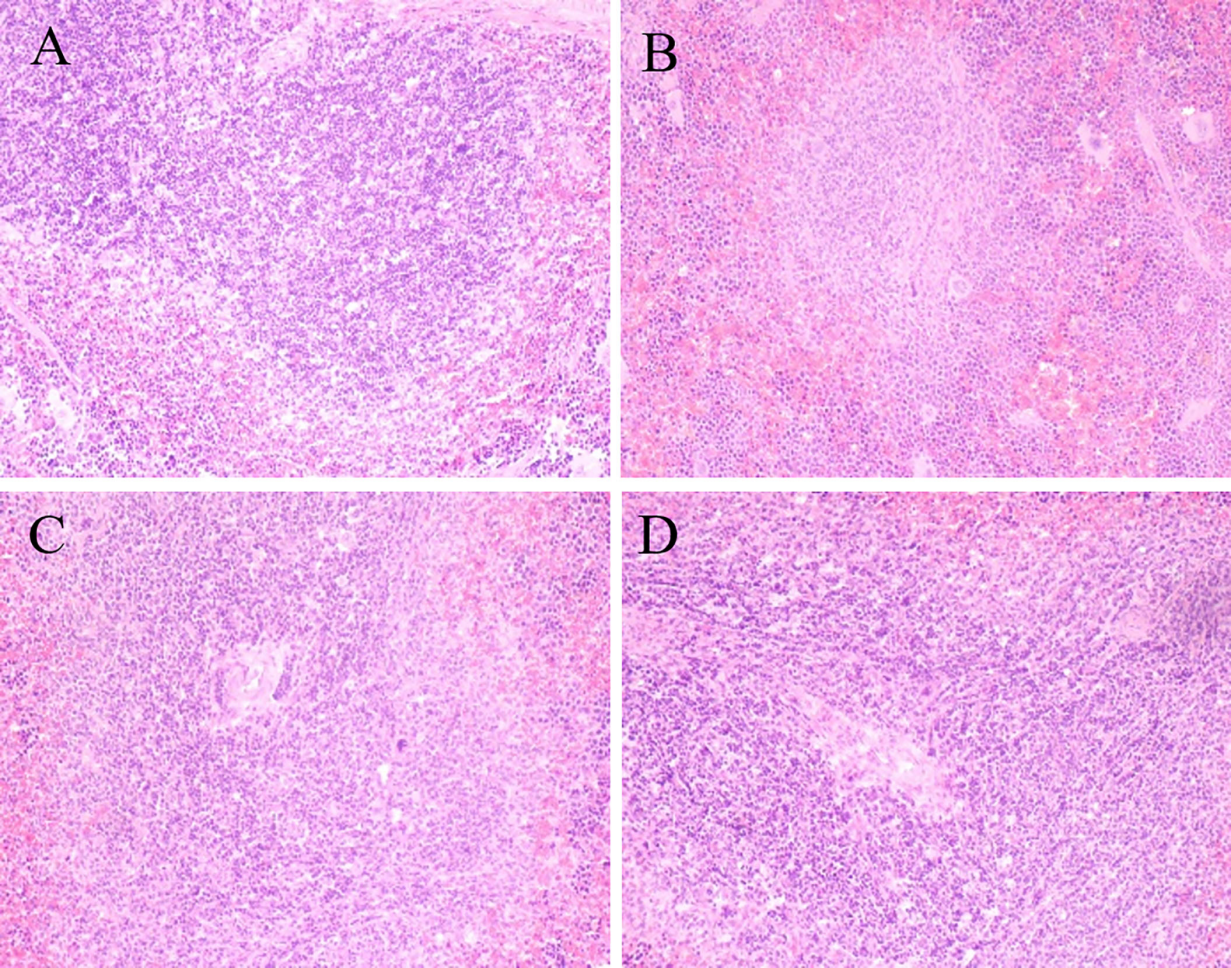
Effects of SPSP on the histopathological changes of the spleen. After SPSP treatment for 14 days, histopathological changes of the spleen were analyzed by image Pro-Plus 7200 software. The samples were HE stained and observed under 200× magnification. **(A)** Normal control group, **(B)** BDS model group, **(C)** L-SPSP (0.1 g/kg) group, **(D)** H-SPSP (0.4 g/kg) group.

In the control group, the splenic trabecular structure was intact, the white pulp of spleen was normal, the central artery was visible, and the splenic sinus had no dilation or congestion. However, in the BDS group, the white pulp of spleen atrophied, the number of lymphocytes reduced, but the number of macrophages increased, and the splenic red pulp was congested. However, after treatment with SPSP or DOL, all the adverse phenomena observed in the BDS group were reversed, and the boundaries between red and white pulps became clear (**Figure 4**).

### Analysis of EPO, G-CSF, TNF-α and IL-6 in Mice Serum

**Figure 5:**
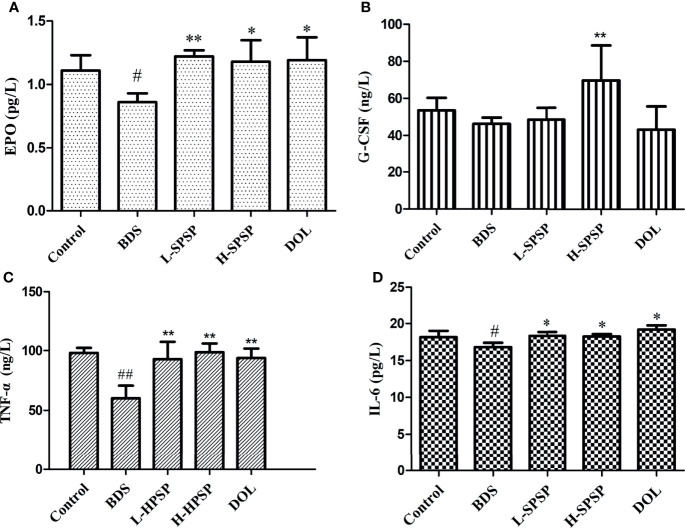
The effect of SPSP on hematopoietic functions **(A, B)** and immunomodulatory cytokines **(C, D)** in BDS mice. **(A)** The level of EPO. **(B)** The level of G-CSF. **(C)** The level of TNF-*α*. **(D)** The level of IL-6. Each value is represented the mean ± SD (n = 3); ^#^ *p* < 0.05, ^##^ *p* < 0.01 versus Control group. ^*^ *p* < 0.05, ^**^ *p* < 0.01 versus BDS.

The effects of SPSP on the levels of hematopoietic and immunomodulatory cytokines in the BDS model mice serum were described in **Figure 5**. The levels of EPO, G-CSF, TNF-*α* and IL-6 in the BDS model group were decreased significantly, when compared to the control group (*p*< 0.01 or *p*< 0.05). Compared with the BDS model group, the levels of the above four cytokines in BDS mice treated with SPSP or DOL were elevated significantly (*p*< 0.01 or *p*< 0.05).

### Spleen Transcriptome Profiling of Control, BDS Model and BDS-SPSP Groups

**Figure 6:**
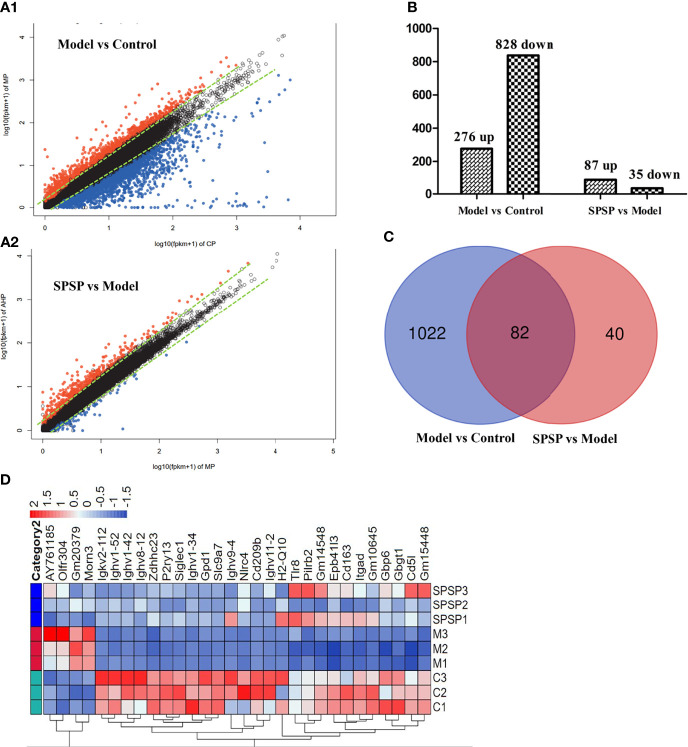
General analysis of DEGs. **(A1)** Volcano plots of DEGs in model (M) vs control **(C)** group. **(A2)** Volcano plots of DEGs in SPSP vs model group. **(B)** Numbers of upregulated and downregulated DEGs. **(C)** Venn diagram of DEGs in M vs C and SPSP vs M comparisons. **(D)** Hierarchical clustering of overlapped DEGs.

To explore potential mechanisms underlying SPSP alleviating BDS, we performed RNA-seq of spleen tissues in the three groups (control, model, and SPSP). DEGs in model vs control group (**Figure 6A1**), and SPSP vs model group (**Figure 6A2**) were visualized using volcano plots. Totally, there were 1104 DEGs which had fold change ≥2 and qValue ≤ 0.05 in BDS spleens compared to the control normal mice, containing 276 up-regulated, and 828 down-regulated genes (**Figure 6B**). Furthermore, 122 DEGs were selected out from SPSP vs model group, of which 87 DEGs were up-regulated and 35 DEGs were down-regulated (**Figure 6B**). Meanwhile, the intersection of 1104 DEGs in the model vs control group and 122 DEGs in the SPSP vs model group were analyzed, there were 82 overlapped DEGs (**Figure 6C**). According to the value of LogFC, a heatmap of the top 30 regulated genes was shown in **Figure 6D**. These results indicated that SPSP could recover the expression level of overlapped DEGs altered by CTX and APH.

### Functional Analysis of DEGs

**Figure 7:**
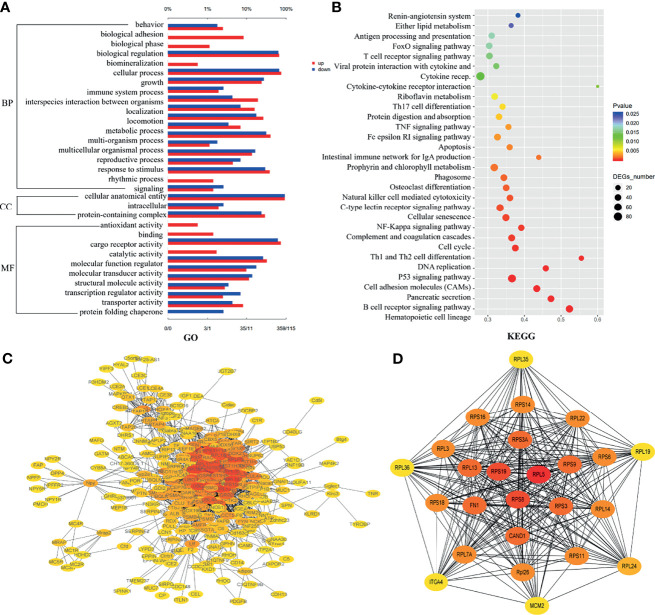
Functional analysis of DEGs. **(A)** List of enriched Go term on BP, CC and MF. **(B)** List of enriched KEGG terms. **(C)** Protein-protein interaction (PPI) network. **(D)** The subset of PPI network. The top 25 DEGs were selected for magnification according to the degree value. The node color was from yellow to red, and the corresponding degree was gradually larger.

The DEGs between SPSP treatment group and model group were analyzed by GO enrichment analysis. Most of DEGs were associated with biological process (BP), only a few were associated with cellular component (CC) and molecular functions (MF). In terms of BP, both the upregulated and downregulated DEGs were associated with the growth and metabolic process of splenocyte, and immune system process (**Figure 7A**). The KEGG pathway enrichment analysis was used to explore a deep insight into gene functions of DEGs (**Figure 7B**). The SPSP-regulated DEGs were significantly related to hematopoietic cell lineage, B cell receptor signaling pathway, P53 and NF-*κB* signaling pathway, Th1 and Th2 cell differentiation, complement and coagulation cascades, leukocyte transendothelial migration, etc. The results indicated that multiple immunoregulatory signaling pathways participated in the treatment of SPSP on BDS. PPI network of overlapped DEGs was showed in **Figure 7C**. The top 25 DEGs were selected out for magnification (**Figure 7D**). RPS19, RPS8 and RPL5 were the top three points with higher degree of connectivity, indicating that they might be the key molecules of SPSP on treating BDS.

### Experiments Validated the Jak1, Stat1, EPOR and GATA1 mRNA Expression in Spleen

**Figure 8:**
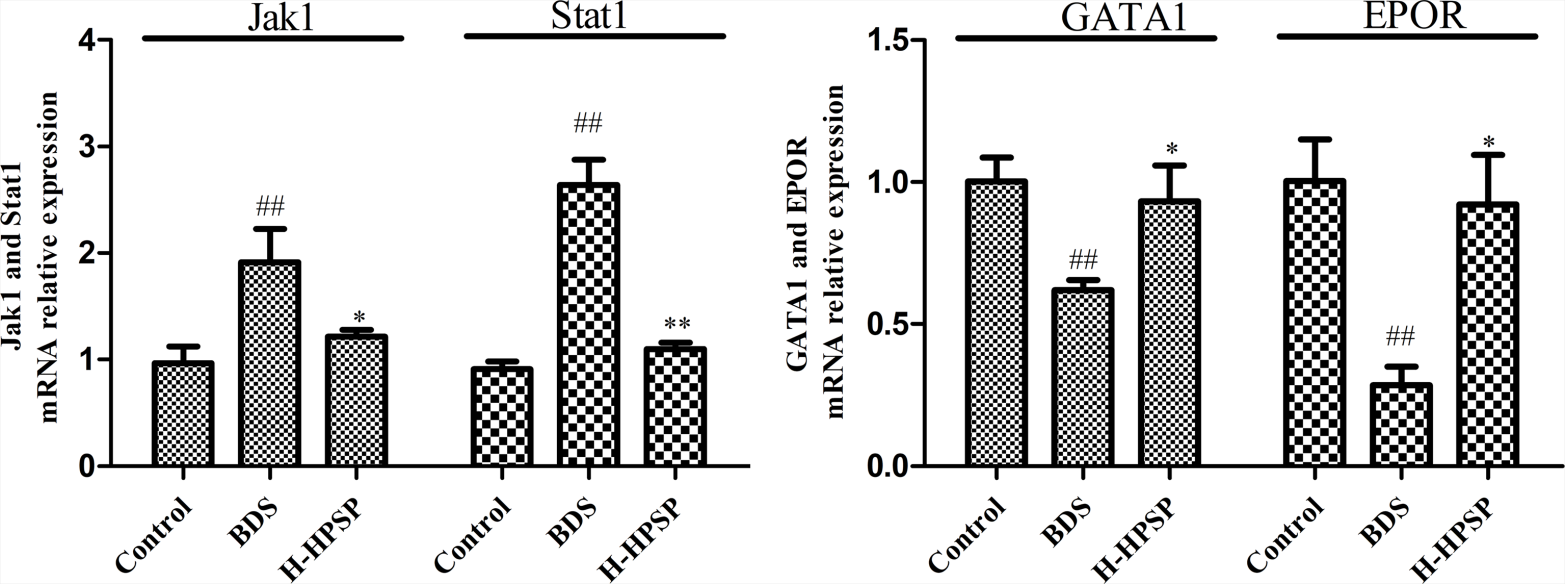
The effects of H-SPSP on mRNA expression of Jak1, Stat1, EPOR and GATA1 in BDS mice. The relative gene expression values were calculated with the 2^−△△Ct^ method. Each value represents the mean ± SD (n = 3); ^##^ *p* < 0.01 versus Control group. ^*^ *p* < 0.05, ^**^ *p* < 0.01 versus BDS.

Janus-activated kinase (JAK)-signal transducer and activator of transcription (STAT) signaling pathway play a key role in regulating many cytokines (EPO, G-CSF, TNF-*α* and IL-6) in the progress of BDS (32). As shown in **Figure 8**, Jak1, STAT1 mRNA expression in spleen of BDS mice were significantly upregulated (*p*<0.01), on the contrary, the expression of EPOR and GATA1 mRNA were significantly downregulated compared to the control group (*p*<0.01). Compared to the BDS model group, the treatment of H-SPSP could downregulated the levels of the Jak1 and STAT1, and upregulated the levels of GATA1 mRNA notably (*p*<0.05 or *p*<0.01).

### Western Blotting Analysis

**Figure 9:**
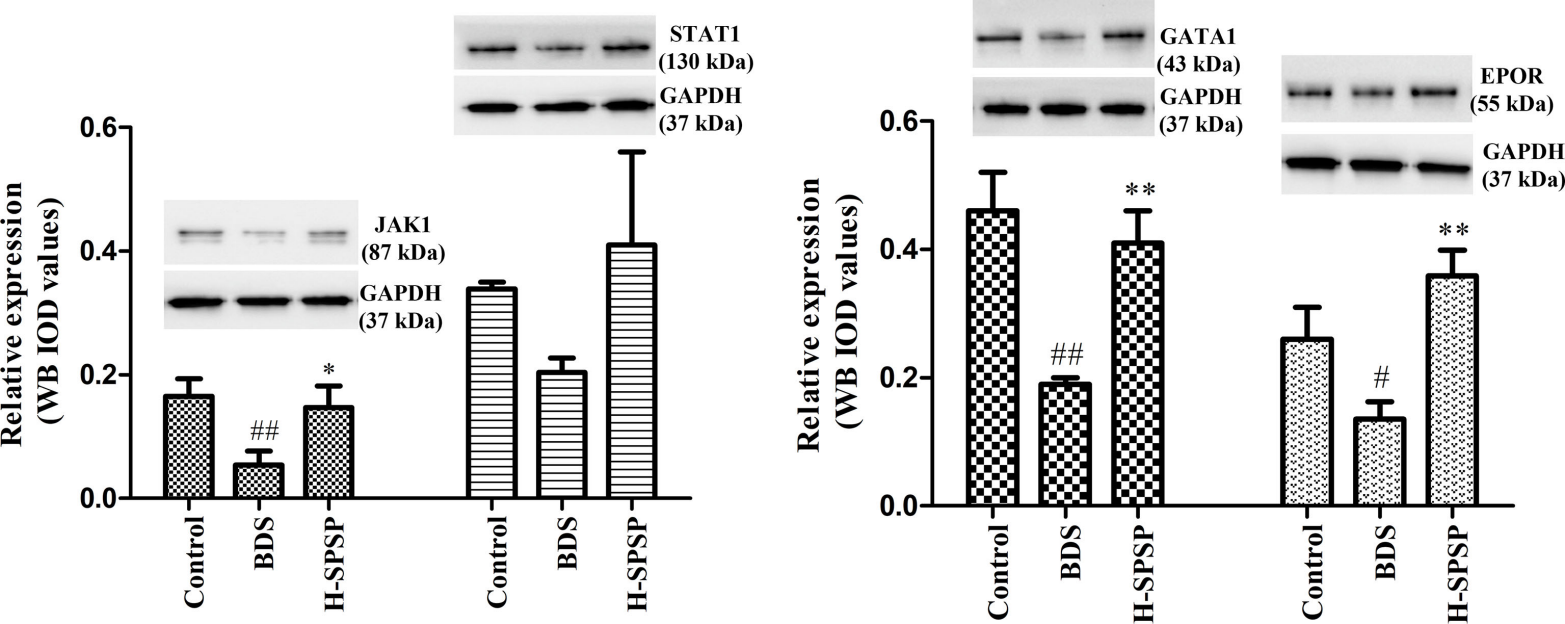
The effects of H-SPSP on protein expression levels of Jak1, Stat1, EPOR and GATA1 in BDS mice. The protein expression levels were normalized to GAPDH and expressed in terms of the fold change relative to the levels in the control group. Each value represents the mean ± SD (n = 3); ^#^ *p* < 0.05, ^##^ *p* < 0.01 versus Control group. ^*^ *p* < 0.05, ^**^ *p* < 0.01 versus BDS.

**Figure 10:**
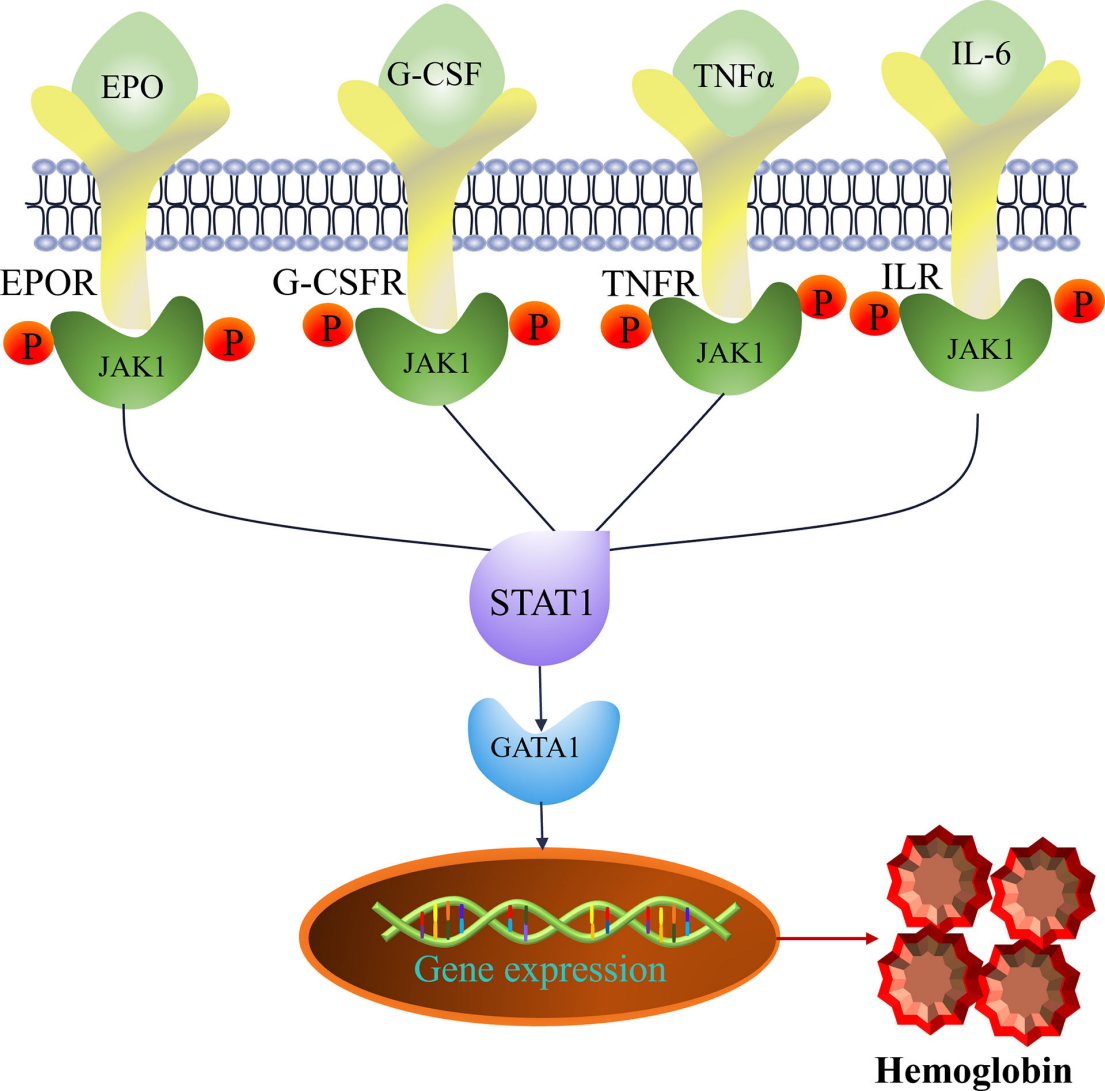
Hematopoietic cytokines (EPO, G-CSF, TNF-*α* and IL-6) and targets (EPOR, JAK1, STAT1 and GATA1) predicted to be involved in the JAK-STAT pathway.

As shown in **Figure 9**, the protein expression levels of JAK1, GATA1, and EPOR in the BDS group were significantly lower than in the control group (*p*<0.05 or *p*<0.01). Compared to the BDS model group, the treatment of H-SPSP significantly increased the protein levels of JAK1, GATA1, and EPOR in BDS mice (*p*<0.05 or *p*<0.01). Whereas, there was no significant difference in the expression of STAT1 among the three groups (*p*>0.05). The potential regulation of H-SPSP on the JAK1-STAT1 signaling pathway in the spleen was shown in **Figure 10**.

## Discussion

In recent years, saccharides from various natural plants have been attracting great attention for practical applications in pharmaceuticals and health-care products because of their low toxicity and multiple biological activities, including anti-tumor, immunomodulatory, hypoglycemic, and antioxidant activities (33). Specifically, PS is an important traditional Chinese medicinal food, and its major bioactive components are exactly polysaccharides. In clinics, PS are mainly used as processed pieces. According to related reports, the type and content of polysaccharide in PS will change after processing (18, 20).

In this study, we focused on the primary structure of SPSP and its regulatory effect in BDS model mice. The results demonstrated that the main components of SPSP were Gal and GalA along with fewer amounts of Man, Glc, Ara, Rha and GlcN in molar ratios of 49.7: 25.6: 12.7:6.2:3.2:2.3:0.3, which differ from the polysaccharides composition of CPSP (Gal, Man, Glu, and Gal A in molar ration of 29.6:36.1:15.1:10.2) and WPSP (Gal, Man, and Gal A in molar ration of 78.8:5.5:13.8) that have been reported (20).

PS processed by different methods have different biological functions, and a structural analysis of SPSP is vital to understand the structure-function relationship (34, 35). Several previous studies showed that processed PS could enhance the effects on benefiting qi, nourishing blood as well as invigorating the spleen and kidney after processing (20, 21). In this study, a BDS mice model established by hypodermic injection of APH and intraperitoneal injection of CTX was used to explore the blood tonification effects of SPSP. After modeling, the BDS mice revealed an obvious change in their appearances, demonstrating mental sluggishness, sleepiness, arched back, movement retardation and weight loss. The blood routine results showed that the peripheral RBC, HGB, and PLT of BDS mice were decreased significantly, which were characteristic clinical manifestations of blood deficiency and anemia (36). However, the number of WBCs in the BDS group was more than the control group, suggesting that CTX and APH led to a compensatory increase in the level of WBCs. After treatment with SPSP, the body temperature of BDS mice returned to normal, and the peripheral blood cell count was also resumed to its normal values. The results indicated that SPSP could promote hematopoietic recovery in BDS mice, verifying its traditional usage of blood tonification.

In recent years, studies have proven that spleen function is closely related to blood deficiency and immunity. Spleen is one of the organs that most likely and first to be affected by anemia, and its enlargement is also a diagnostic feature for anemia (37, 38). The accumulation of bone marrow progenitor cells and RBCs could enlarge the spleen, which could in turn exacerbate the symptoms of anemia (39, 40). In the present study, the spleen index in the BDS group increased remarkably, and the number of RBCs dropped notably. On the other hand, the spleen index and RBCs in mice treated with SPSP were significantly reversed compared to those of the BDS group. Moreover, the result of histopathological changes in the spleen showed that SPSP could prevent the atrophy of the spleen, and restore all of structural features of the spleen to normal. It indicated that SPSP could alleviate BDS induced by CTX and APH, thereby protecting the immune organ.

A series of hematopoietic-related cytokines, such as GM-CSF, G-CSF, EPO, IL-3, and IL-6, participate in blood cell formation (41, 42). Specifically, EPO is an important glycoprotein hormone in the regulation of erythropoiesis. Besides, G-CSF promotes erythroblast survival until they become erythrocytes with impaired cellular functions (42). G-CSF and EPO showed good synergistic *in vivo* and *in vitro* effects on anemia alleviation (43–45). In the results reported herein, the blood EPO and G-CSF levels in the BDS group were lower than the control group but increased significantly upon SPSP administration, indicating that SPSP might alleviate hematopoietic damage by promoting EPO and G-CSF production. Blood deficiency is often accompanied by impaired immune functions. Immune cytokines such as TNF-*α*, IFN-*γ*, IL-3 and IL-6 play essential roles in the coordination and intercellular communication of lymphoid cells, inflammatory cells, and hematopoietic cells in the immune system (46). In this study, the BDS group revealed significantly lower levels of serum TNF-*α* and IL-6 compared to the control group, and such an inhibitory effect of BDS was reversed with SPSP, indicating that SPSP might alleviate blood deficiency by enhancing immune functions.

Nowadays, RNA-seq studies have provided insight into the molecular mechanisms of anemia, and the mechanisms of traditional Chinese medicine against anemia or BDS. Transcriptome analysis indicated that P53 dependent pathway, hematopoiesis, vascular development and apoptosis played vital role in pathology of diamond–blackfan anemia (47). RNA-seq study of whole blood showed that inflammatory response, hematopoiesis, lymphocyte function, coagulation and red blood cell regeneration were associated with the immune-mediated hemolytic anemia (48). E’jiao had been used to nourishing blood in China for more than two thousand years. RNA-seq revealed that the mechanisms of E’jiao were associated with hematopoietic cell lineage, ECM-receptor interaction, osteoclast differentiation, etc (6).

Similar to the previous studies, the DEGs were found significantly enriched in hematopoietic cell lineage, B cell receptor signaling pathway, P53 signaling pathway, NF-*κ*B signaling pathway, Th1 and Th2 cell differentiation, complement and coagulation cascades, cell adhesion molecules (CAMs), osteoclast differentiation, etc in the present study. Our data indicated that the medicate action of SPSP on BDS were through regulating hematopoietic cell lineage and multiple immune-related signaling pathways (B cell receptor signaling pathway, P53 signaling pathway, Th1 and Th2 cell differentiation, etc). This result was also consistent with the regulation of SPSP on hematopoietic function factors (EPO, G-CSF) and inflammatory cytokines (IL-6, TNF-*α*).

JAK-STAT signaling pathway was demonstrated to be one of the critical pathways in transduction of cytokines signals involved in hematopoiesis, immune regulation and inflammation (49, 50). The binding of EPO and its receptor EPOR could rapidly activate the JAK-STAT signaling pathway, and promoted the survival, proliferation and differentiation of EP cells by upregulating erythroid-specific transcription factor GATA1 (51, 52). In this pathway, Jak1, Stat1, GATA1and EPOR were the major targets to treat BDS (7). Danggui Buxue Tang could significantly attenuated the levels of key targets (Jak1, Stat1 and Stat3) of the Jak/Stat signaling pathway in immune-induced aplastic anemia mice (53). Jujube polysaccharides have been showed the improvement on chronic kidney disease-associated anemia by alternating the serum EPO level, kidney EPO mRNA and protein expression (54). Steamed *Panax notoginseng* was reported to attenuate BDS *via* up-regulating GATA1 mRNA expression (7). CTX is widely used to induce immunosuppression in animal model for its inhibiting activities on humoral and cellular immunity (55, 56). It had been demonstrated that *Polygonatum sibiricum* polysaccharide had an immune boosting effect on CTX-induced immunosuppression in chickens by promoting the humoral immunity, cellular immunity, and protecting immune organs (57). The hematopoietic stem cells in the marrow and peripheral blood cells could deplete by APH, which resulting in hematopoietic and immune suppression (58). As shown in this study, the expression of Jak1, GATA1, EPOR proteins, and GATA1, EPOR mRNA’s were significantly inhibit by the injection of APH and CTX. However, the treatment of APH and CTX caused a feedback increase in the mRNA expression of Jak1 and Stat1, which was a compensatory mechanism. The treatment of SPSP can antagonize CTX-induced decrease protein levels of Jak1, Stat1, GATA1, and EPOR, and the upregulate mRNA levels of GATA1 and EPOR, which was also consistent with the improved routine blood indexes, hematopoietic and inflammatory cytokines (IL-6, TNF-α) in this study. Therefore, we speculate that the hematinic function of SPSP on BDS mice might be through regulating the key molecules in JAK1-STAT1 signaling pathway.

## Conclusion

It was the first time to isolate and purify polysaccharides from the steam-processing of *P. sibiricum*. The results suggested that SPSP was consisted of Man, Glc, Ara, Rha and GlcN. Furthermore, SPSP exhibited the hematopoietic effect on BDS mice by reversing the abnormal decrease of peripheral blood cells, preventing spleen enlargement, and regulating the levels of hematopoiesis and immune cytokines. In addition, RNA-seq analysis elucidated that the potential molecular mechanisms of the blood-enriching effects of SPSP were mainly involved in hematopoiesis, immune regulation signaling pathways. Experimental verification indicated that the effect of SPSP on BDS might be regulation of JAK1-STAT1 pathway, and elevated the hematopoietic cytokines (EPO, G-CSF, TNF-*α* and IL-6). Our investigations suggested that SPSP might be a potent immunomodulator to prevent and treat blood deficiency.

## Data Availability Statement

The datasets presented in this study can be found in online repositories. The names of the repository/repositories and accession number(s) can be found below: https://www.ncbi.nlm.nih.gov/, PRJNA778421.

## Ethics Statement

The animal study was reviewed and approved by Animal Ethical and Experimental Committee of the Zhejiang Pharmaceutical College.

## Author Contributions

JW drafted and participated in preparing all figures and manuscript. FW participated in data analysis and some part of manuscript preparation. LY and HR provided intellectual and data analysis support. XP designed RNA-Seq analysis. ZZ, XF, and LZ performed the extraction and structure identification of steam-processed *Polygonatum sibiricum* polysaccharide. All authors contributed to the article and approved the submitted version.

## Funding

This work was supported financially by General scientific research project of Zhejiang education department (Y202044666), Scientific Research Program of Yiwu, China (20-3-124), Zhejiang Institute of Drug Supervision and Administration and Industrial Development (ZYH2020004).

## Conflict of Interest

Authors of ZZ, XF, and LZ are employed by Zhejiang Sanxitang Chinese Medicine Co., LTD.

The remaining authors declare that the research was conducted in the absence of any commercial or financial relationships that could be construed as a potential conflict of interest.

## Publisher’s Note

All claims expressed in this article are solely those of the authors and do not necessarily represent those of their affiliated organizations, or those of the publisher, the editors and the reviewers. Any product that may be evaluated in this article, or claim that may be made by its manufacturer, is not guaranteed or endorsed by the publisher.

## References

[1] Li PL Sun HG Hua YL Ji P Zhang L Li JX. Metabolomics Study of Hematopoietic Function of Angelica Sinensis on Blood Deficiency Mice Model. J Ethnopharmacol (2015) 166:261–9. doi: 10.1016/j.jep.2015.03.010 25797116

[2] Shi XQ Zhu ZH Yue SJ Tang YP Chen YY Pu ZJ. Integration of Organ Metabolomics and Proteomics in Exploring the Blood Enriching Mechanism of Danggui Buxue Decoction in Hemorrhagic Anemia Rats. J Ethnopharmacol (2020) 261:113000. doi: 10.1016/j.jep.2020.113000 32663590

[3] Park EJ Baek SE Kim M Kim AR Park HJ Kwon O. Effects of Herbal Medicine (Danggwijagyaksan) for Treating Climacteric Syndrome With a Blood-Deficiency-Dominant Pattern: A Randomized, Double-Blind, Placebo-Controlled Pilot Trial. Integr Med Res (2021) 10:100715. doi: 10.1016/j.imr.2021.100715 33665100 PMC7903340

[4] Zhang H Wang HF Liu Y Huang LJ Wang ZF Li Y. The Haematopoietic Effect of Panax Japonicus on Blood Deficiency Model Mice. J Ethnopharmacol (2014) 154:818–24. doi: 10.1016/j.jep.2014.05.008 24837302

[5] Akpinar H Cetiner M Keshav S Ormeci N Toruner M. Diagnosis and Treatment of Iron Deficiency Anemia in Patients With Inflammatory Bowel Disease and Gastrointestinal Bleeding: Iron Deficiency Anemia Working Group Consensus Report. Turk J Gastroenterol (2017) 28:81–7. doi: 10.5152/tjg.2017.17593 28119272

[6] Zhang Y Ye TT Gong SQ Hong ZP Zhou XS Liu HB. RNA-Sequencing Based Bone Marrow Cell Transcriptome Analysis Reveals the Potential Mechanisms of E'jiao Against Blood-Deficiency in Mice. BioMed Pharmacother (2019) 118:109291. doi: 10.1016/j.biopha.2019.109291 31401395

[7] Zhang ZJ Zhang YM Gao M Cui XM Yang Y Duijn BV. Steamed Panax Notoginseng Attenuates Anemia in Mice With Blood Deficiency Syndrome *via* Regulating Hematopoietic Factors and JAK-STAT Pathway. Front Pharmacol (2020) 10:1578. doi: 10.3389/fphar.2019.01578 32038252 PMC6985777

[8] Ji P Wei YM Hua YL Zhang XS Yao WL Ma Q. A Novel Approach Using Metabolomics Coupled With Hematological and Biochemical Parameters to Explain the Enriching-Blood Effect and Mechanism of Unprocessed Angelica Sinensis and its 4 Kinds of Processed Products. J Ethnopharmacol (2018) 211:101–16. doi: 10.1016/j.jep.2017.09.028 28958590

[9] He Y Gao TH Li J Chen ZJ Wang LJ Zhang JM. Metabonomics Study on the Effect of Siwu Decoction for Blood Deficiency Syndrome in Rats Using UPLC-Q/TOF-MS Analysis. BioMed Chromatogr (2019) 33:e4617. doi: 10.1002/bmc.4617 31207665

[10] Committee for the Pharmacopoeia of PR China. Pharmacopoeia of PR China (Part I). Beijing: Chinese Medicine Science and Technology Publishing House (2020). p. 319.

[11] Liu N Dong ZH Zhu XS Xu HY Zhao ZX. Characterization and Protective Effect of Polygonatum Sibiricum Polysaccharide Against Cyclophosphamide-Induced Immunosuppression in Balb/c Mice. Int J Biol Macromol (2018) 107:796–802. doi: 10.1016/j.ijbiomac.2017.09.051 28939510

[12] Long T Liu Z Shang J Zhou X Yu S Tian H. Polygonatum Sibiricum Polysaccharides Play Anti-Cancer Effect Through TLR4-MAPK/NF-kappaB Signaling Pathways. Int J Biol Macromol (2018) 111:813–21. doi: 10.1016/j.ijbiomac.2018.01.070 29343453

[13] Cui X Wang S Cao H Guo H Li Y Xu F. The Bioactivities and Pharmacological Applications of Polygonatum Sibiricum Polysaccharides. Molecules (2018) 23:1170. doi: 10.3390/molecules23051170 PMC6099637 29757991

[14] Li L Thakur K Liao BY Zhang JG Wei ZJ. Antioxidant and Antimicrobial Potential of Polysaccharides Sequentially Extracted From Polygonatum Cyrtonema Hua. Int J Biol Macromol (2018) 114:317–23. doi: 10.1016/j.ijbiomac.2018.03.121 29578016

[15] Yelithao K Surayot U Park WJ Lee SM Lee DH You SG. Effect of Sulfation and Partial Hydrolysis of Polysaccharides From *Polygonatum Sibiricum* on Immune-Enhancement. Int J Biol Macromol (2019) 122:10–8. doi: 10.1016/j.ijbiomac.2018.10.119 30336240

[16] Wang Y Lan CJ Liao X Chen D Song WG Zhang QL. Polygonati Sibiricum Polysaccharide Potentially Attenuate Diabetic Retinal Injury in a Diabetic Rat Model. J Diabetes Investig (2019) 10:915–24. doi: 10.1111/jdi.12976 PMC6626950 30426692

[17] Liu TY Zhao LL Chen SB Hou BC Huang J Hong X. Polygonatum Sibiricum Polysaccharides Prevent LPS−induced Acute Lung Injury by Inhibiting Inflammation *via* the TLR4/Myd88/NF−κb Pathway. Exp Ther Med (2020) 20:3733–9. doi: 10.3892/etm.2020.9097 PMC7444378 32855724

[18] Zhang YX Zhou Q Zhang XL Li HF Wu P Zhang ZH. Content Change and Transformation Mechanism of Oligosaccharides and Monosaccharides Before and After Processing in Polygonatum Cyrtonema. Zhong Yao Cai (2020) 43:318–22. doi: 10.13863/j.issn1001-4454.2020.02.012

[19] Gan XF Wei GL Li TT Qu ZY Xu S He M. Study on Content of Polysaccharide During Processing of Polygonatum Sibiricum by RSM and PMP-HPLC Characteristic Map Technology. Zhong Cao Yao (2019) 50:4932–41. doi: 10.7501/j.issn.0253-2670.2019.20.014

[20] Sun TT Zhang H Li Y Liu Y Dai W Fang J. Physicochemical Properties and Immunological Activities of Polysaccharides From Both Crude and Wine-Processed Polygonatum Sibiricum. Int J Biol Macromol (2020) 143:255–64. doi: 10.1016/j.ijbiomac.2019.11.166 31760031

[21] Ma MN Dong YJ Lei SS Wan XQ Lv GY Chen SH. Pharmacodynamic Effects of Different Processed Products of Rhizoma Polygonati on Rat Model of Qi and Yin Deficiency. SH.J.TCM (2019) 53:83–9. doi: 10.16305/j.1007-1334.2019.10.020

[22] Lin Y She L Wei XY Tang ML Jiang SD Qu Y. Study on Chemical Constituents, Detoxification and Synergism of *Polygonati Rhizoma* Before and After Processing. Zhong Yao Cai (2021) 44(6):1353–9. doi: 10.13863/j.issn1001-4454.2021.06.012

[23] Jia L Yu JD He L Wang HX Jiang LL Miao XY. Nutritional Support in the Treatment of Aplastic Anemia. Nutrition (2011) 27:1194–201. doi: 10.1016/j.nut.2011.01.012 21621387

[24] Li WX Tang YP Guo JM Huang MY Li W Qian DW. Enriching Blood Effect Comparison in Three Kinds of Blood Deficiency Model After Oral Administration of Drug Pair of Angelicae Sinensis Radix and Chuanxiong Rhizoma and Each Single Herb. Zhongguo Zhong Yao Za Zhi (2011) 36:1808–14. 22032150

[25] Zhao P Li X Wang Y Yan LY Guo LP Huang LQ. Characterisation and Saccharide Mapping of Polysaccharides From Four Common Polygonatum Spp. Carbohydr Polymers (2020) 1:115836. doi: 10.1016/j.carbpol.2020.115836 32059888

[26] Ru Y Chen X Wang J Guo LH Lin ZY Peng X. Structural Characterization, Hypoglycemic Effects and Mechanism of a Novel Polysaccharide From Tetrastigma Hemsleyanum Diels Et Gilg. Int J Biol Macromol (2019) 123:775–83. doi: 10.1016/j.ijbiomac.2018.11.085 30447363

[27] Robinson MD McCarthy DJ Smyth GK. Edger: A Bioconductor Package for Differential Expression Analysis of Digital Gene Expression Data. Bioinformatics (2010) 26:139–40. doi: 10.1093/bioinformatics/btp616 PMC2796818 19910308

[28] Hu DJ Cheong KL Zhao J Li SP. Chromatography in Characterization of Polysaccharides From Medicinal Plants and Fungi. J Sep Sci (2013) 36:1–19. doi: 10.1002/jssc.201200874 23225747

[29] Wang YJ Liu N Xue X Li Q Sun DQ Zhao ZX. Purification, Structural Characterization and *In Vivo* Immunoregulatory Activity of a Novel Polysaccharide From Polygonatum Sibiricum. Int J Biol Macromol (2020) 160:688–94. doi: 10.1016/j.ijbiomac.2020.05.245 32479947

[30] Xu Y Fei C Yu Z Ling Z Li X Yu Y. Optimisation of Pressurised Water Extraction of Polysaccharides From Blackcurrant and its Antioxidant Activity. Food Chem (2016) 194:650–8. doi: 10.1016/j.foodchem 26471604

[31] Tian BR Li WR Wang J Liu YM. Functional Polysaccharide-Based Film Prepared From Chitosan and *β*-Acids: Structural, Physicochemical, and Bioactive Properties. Int J Biol Macromol (2021) 181:966–77. doi: 10.1016/j.ijbiomac.2021.04.100 33887287

[32] Zhou B Damrauer JS Bailey ST Hadzic T Jeong Y Clark K. Erythropoietin Promotes Breast Tumorigenesis Through Tumor-Initiating Cell Self-Renewal. J Clin Invest (2014) 124:553–63. doi: 10.1172/JCI69804 PMC3904607 24435044

[33] Yang WF Wang Y Li XP Yu P. Purification and Structural Characterization of Chinese Yam Polysaccharide and its Activities. Carbohydr Polym (2015) 117:1021–7. doi: 10.1016/j.carbpol.2014.09.082 25498730

[34] Ferreira SS Passos CP Madureira P Vilanova M Coimbra MA. Structure-Function Relationships of Immunostimulatory Polysaccharides: A Review. Carbohydr Polymers (2015) 132:378–96. doi: 10.1016/j.carbpol.2015.05.079 26256362

[35] Wang X Zhang Z Zhao M. Carboxymethylation of Polysaccharides From Tremella Fuciformis for Antioxidant and Moisture-Preserving Activities. Int J Biol Macromol (2015) 72:526–30. doi: 10.1016/j.ijbiomac.2014.08.045 25194971

[36] Sun J Zhang L He YJ Zhang K Wu LP Fan YS. To Unveil the Molecular Mechanisms of Qi and Blood Through Systems Biology-Based Investigation Into Si-Jun-Zi-Tang and Si-Wu-Tang Formulae. Sci Rep (2016) 6:34328. doi: 10.1038/srep34328 27677604 PMC5039637

[37] Dhaliwal G Cornett PA Tierney LM. Hemolytic Anemia. Am Fam Physician (2004) 69:2599–606. 15202694

[38] Alsalem AH. Splenic Complications of Sickle Cell Anemia and the Role of Splenectomy. Isrn Hematol (2011) 2011:1–7. doi: 10.5402/2011/864257 PMC3200071 22084706

[39] Queiroz ML Valadares MC Bincoletto C Dieamant GC. Ehrlich Ascites Tumor as a Tool in the Development of Compounds With Immunomodulatory Properties. Immunopharmacol Immunotoxicol (2004) 26:511–25. doi: 10.1081/iph-200042289 15658602

[40] Gaspar BL Sharma P Das R. Anemia in Malignancies: Pathogenetic and Diagnostic Considerations. Hematology (2015) 20:18–25. doi: 10.1179/1607845414Y.0000000161 24666207

[41] Sieff CA. Hematopoietic Growth Factors. J Clin Invest (1987) 79:1549–57. doi: 10.1172/JCI112988 PMC424464 3034976

[42] Nikpour M Pellagatti A Liu A Karimi M Malcovati L Gogvadze V. Gene Expression Profiling of Erythroblasts From Refractory Anaemia With Ring Sideroblasts (RARS) and Effects of G-CSF. Br J Haematol (2010) 149:844–54. doi: 10.1111/j.1365-2141.2010.08174.x 20408843

[43] Tehranchi R Fadeel B Forsblom AM Christensson B Samuelsson J Zhivotovsky B. Granulocyte Colony-Stimulating Factor Inhibits Spontaneous Cytochromec Release and Mitochondria-Dependent Apoptosis of Myelodysplastic Syndrome Hematopoietic Progenitors. Blood (2003) 101:1080–6. doi: 10.1182/blood-2002-06-1774 12393561

[44] Tehranchi R Invernizzi R Grandien A Zhivotovsky B Fadeel B Forsblom AM. Aberrant Mitochondrial Iron Distribution and Maturation Arrest Characterize Early Erythroid Precursors in Low-Risk Myelodysplastic Syndromes. Blood (2005) 106:247–53. doi: 10.1182/blood-2004-12-4649 15755901

[45] Jadersten M Montgomery SM Dybedal I Porwit-MacDonald A Hellstrom-Lindberg E. Long-Term Outcome of Treatment of Anemia in MDS With Erythropoietin and G-CSF. Blood (2005) 106:803–11. doi: 10.1182/blood-2004-10-3872 15840690

[46] Mei YX Chen HX Zhang J Zhang XD Liang YX. Protective Effect of Chitooligosaccharides Against Cyclophosphamide-Induced Immunosuppression in Mice. Int J Biol Macromol (2013) 62:330–5. doi: 10.1016/j.ijbiomac.2013.09.038 24080320

[47] Wan Y Zhang Q Zhang ZJ Song BF Wang XM Zhang YC. Transcriptome Analysis Reveals a Ribosome Constituents Disorder Involved in the RPL5 Downregulated Zebrafish Model of Diamond-Blackfan Anemia. BMC Med Genomics (2016) 9:13. doi: 10.1186/s12920-016-0174-9 26961822 PMC4785739

[48] Borchert C Herman A Roth M Brooks AC Friedenberg SG. RNA Sequencing of Whole Blood in Dogs With Primary Immune-Mediated Hemolytic Anemia (IMHA) Reveals Novel Insights Into Disease Pathogenesis. PloS One (2020) 15:e0240975. doi: 10.1371/journal.pone.0240975 33091028 PMC7580939

[49] Vainchenker W Constantinescu SN. JAK/STAT Signaling in Hematological Malignancies. Oncogene (2013) 32:2601–13. doi: 10.1038/onc.2012.347 22869151

[50] Chen L Teng H Fang T Xiao JB. Agrimonolide From Agrimonia Pilosa Suppresses Inflammatory Responses Through Down-Regulation of COX-2/iNOS and Inactivation of NF-kB in Lipopolysaccharide-Stimulated Macrophages. Phytomedicine (2016) 23:846–55. doi: 10.1016/j.phymed.2016.03.016 27288920

[51] Jelkmann W. Molecular Biology of Erythropoietin. Intern Med (2004) 43:649–59. doi: 10.2169/internalmedicine.43.649 15468961

[52] Weiss MJ Orkin SH. Transcription Factor GATA-1 Permits Survival and Maturation of Erythroid Precursors by Preventing Apoptosis. Proc Natl Acad Sci USA (1995) 92:9623–7. doi: 10.1073/pnas.92.21.9623 PMC40854 7568185

[53] Deng PY Li X Wei Y Liu J Chen M Xu YM. The Herbal Decoction Modified Danggui Buxue Tang Attenuates Immune-Mediated Bone Marrow Failure by Regulating the Differentiation of T Lymphocytes in an Immune-Induced Aplastic Anemia Mouse Model. PloS One (2017) 12:e0180417. doi: 10.1371/journal.pone.0180417 28683082 PMC5500321

[54] Huang SY Jiang X Chen QG Hu ZL Wang FC Zhao Y. Jujube Polysaccharides Mitigated Anemia in Rats With Chronic Kidney Disease: Regulation of Short Chain Fatty Acids Release and Erythropoietin Production. J Funct Foods (2021) 86:104673. doi: 10.1016/j.jff.2021.104673

[55] Motoyoshi Y Kaminoda K Saitoh O Hamasaki K Nakao K Ishii N. Different Mechanisms for Anti-Tumor Effects of Low and High-Dose Cyclophosphamide. Oncol Rep (2006) 16:141–6. doi: 10.3892/or.16.1.141 16786137

[56] Kim JW Choi J Seol DJ Choung JJ Ku SK. Immunomodulatory Effects Kuseonwangdogo-Based Mixed Herbal Formula Extracts on a Cyclophosphamide Induced Immunosuppression Mouse Model. Evid Based Complement Altern Med (2018) 2018:6017412. doi: 10.1155/2018/6017412 PMC5911329 29849713

[57] Shu G Xu D Zhao J Yin LZ Lin JC Fu HL. Protective Effect of *Polygonatum Sibiricum* Polysaccharide on Cyclophosphamide-Induced Immunosuppression in Chickens. Res Vet Sci (2021) 135:96–105. doi: 10.1016/j.rvsc.2020.12.025 33461120

[58] Li WX Tang YP Guo JM Shang EX Qian YF Wang LY. Comparative Metabolomics Analysis on Hematopoietic Functions of Herb Pair Gui-Xiong by Ultrahigh-Performance Liquid Chromatography Coupled to Quadrupole Time-of-Flight Mass Spectrometry and Pattern Recognition Approach. J Chromatogr A (2014) 1346:49–56. doi: 10.1016/j.chroma.2014.04.042 24794940

