# Supplementary figures and images for: Blood-Enriching Effects and Immune-Regulation Mechanism of Steam-Processed Polygonatum Sibiricum Polysaccharide in Blood Deficiency Syndrome Mice
Source: Front Immunol. 2022 Feb 17;13:813676. doi: 10.3389/fimmu.2022.813676 (PMC8892585; doi:10.3389/fimmu.2022.813676)

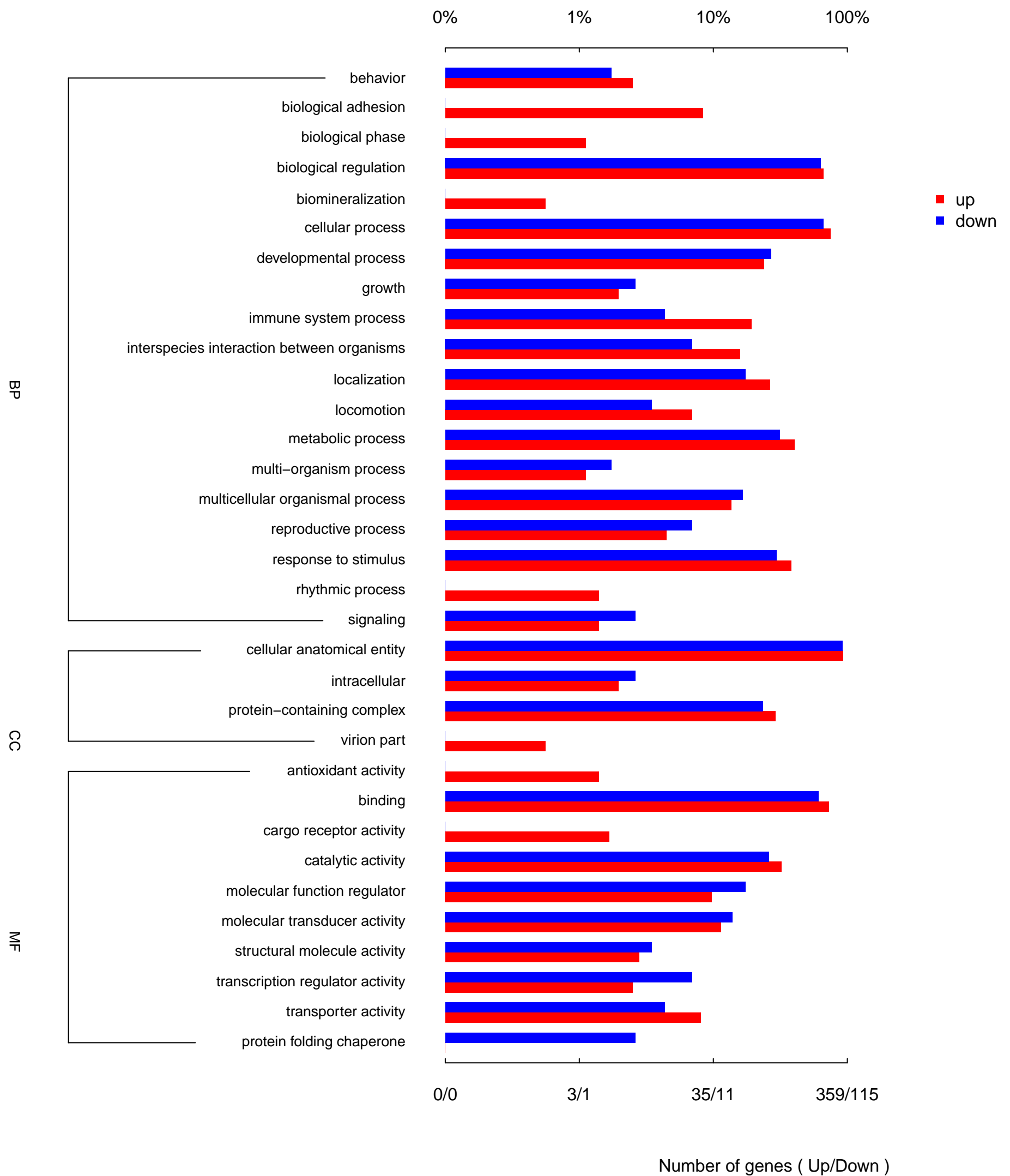

Supplement: Supplementary file 1 [file DataSheet_1.zip › Figure7/GO/SPSP_vs_M.DEG.gobars.pdf]

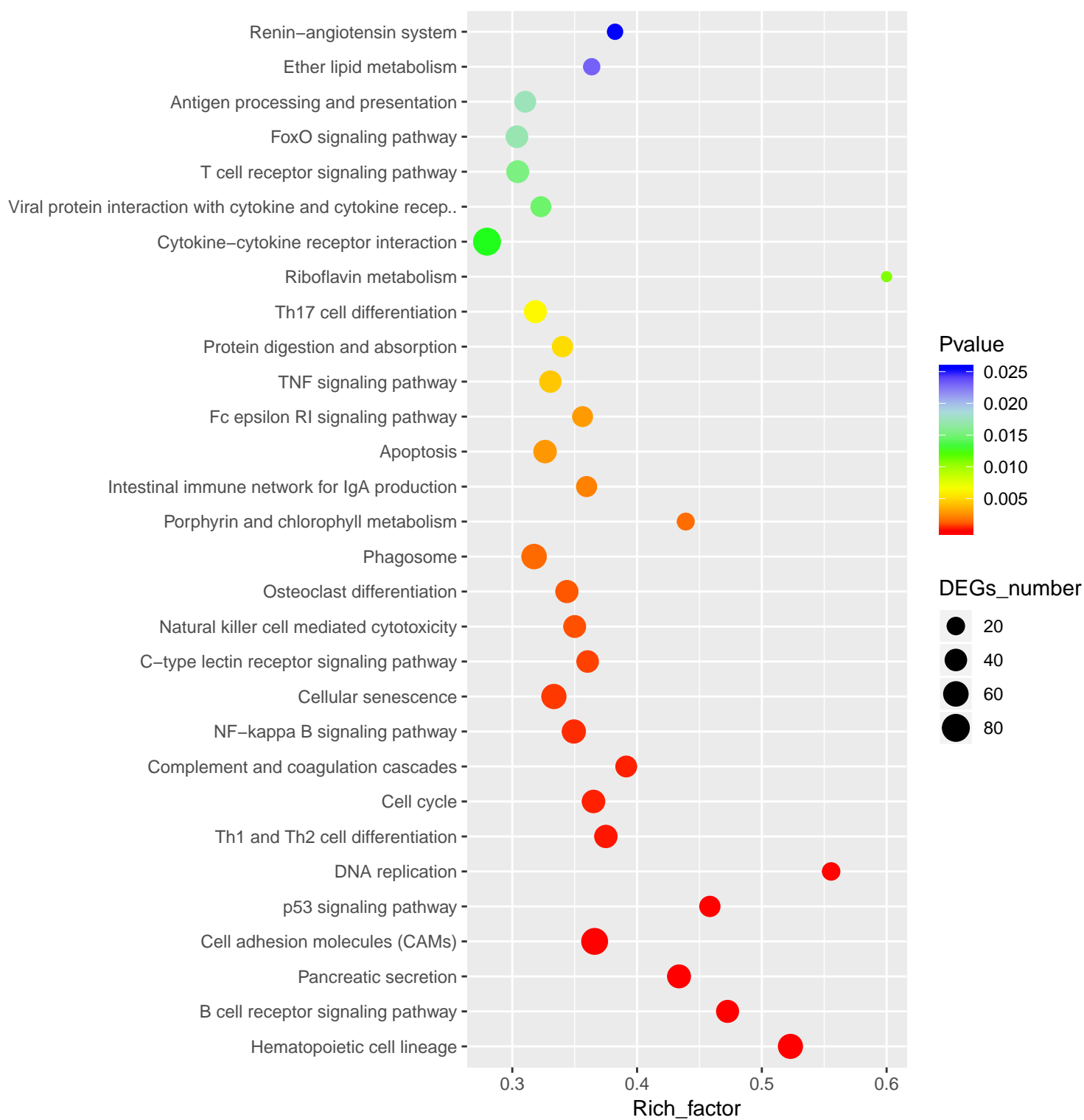

Supplement: Supplementary file 1 [file DataSheet_1.zip › Figure7/KEGG/SPSP_vs_M.DEG.Ko.enrich.pdf]

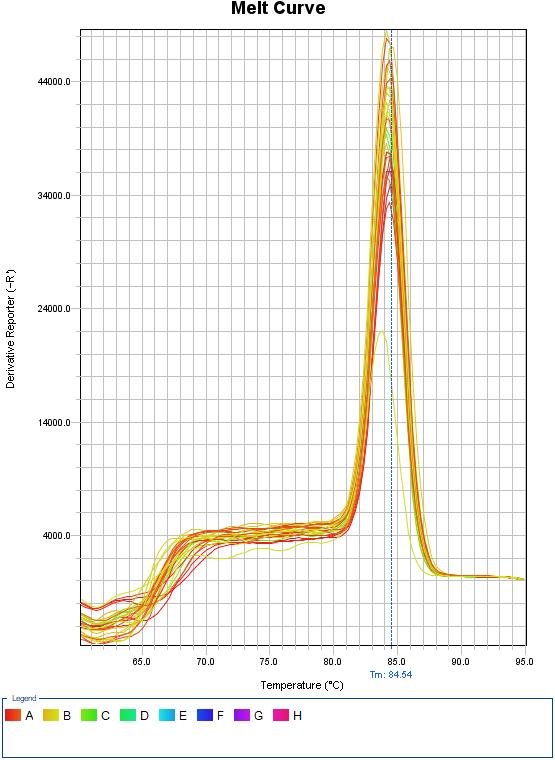

Supplement: Supplementary file 1 [file DataSheet_1.zip › Figure8/Melt Curve-all-EPOR.jpg]

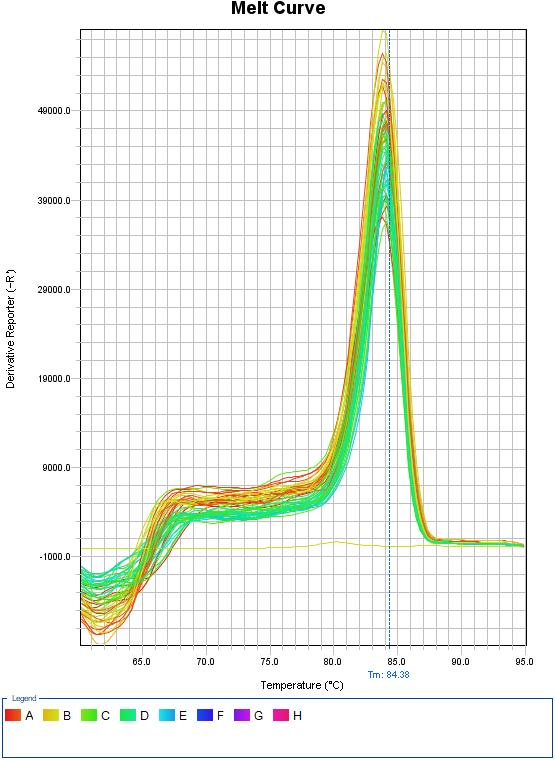

Supplement: Supplementary file 1 [file DataSheet_1.zip › Figure8/Melt Curve-GAPDH.jpg]

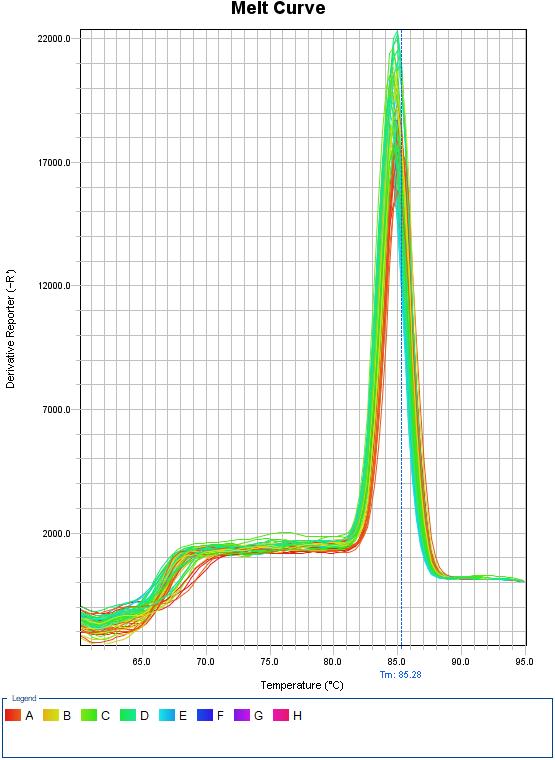

Supplement: Supplementary file 1 [file DataSheet_1.zip › Figure8/Melt Curve-GATA1.jpg]

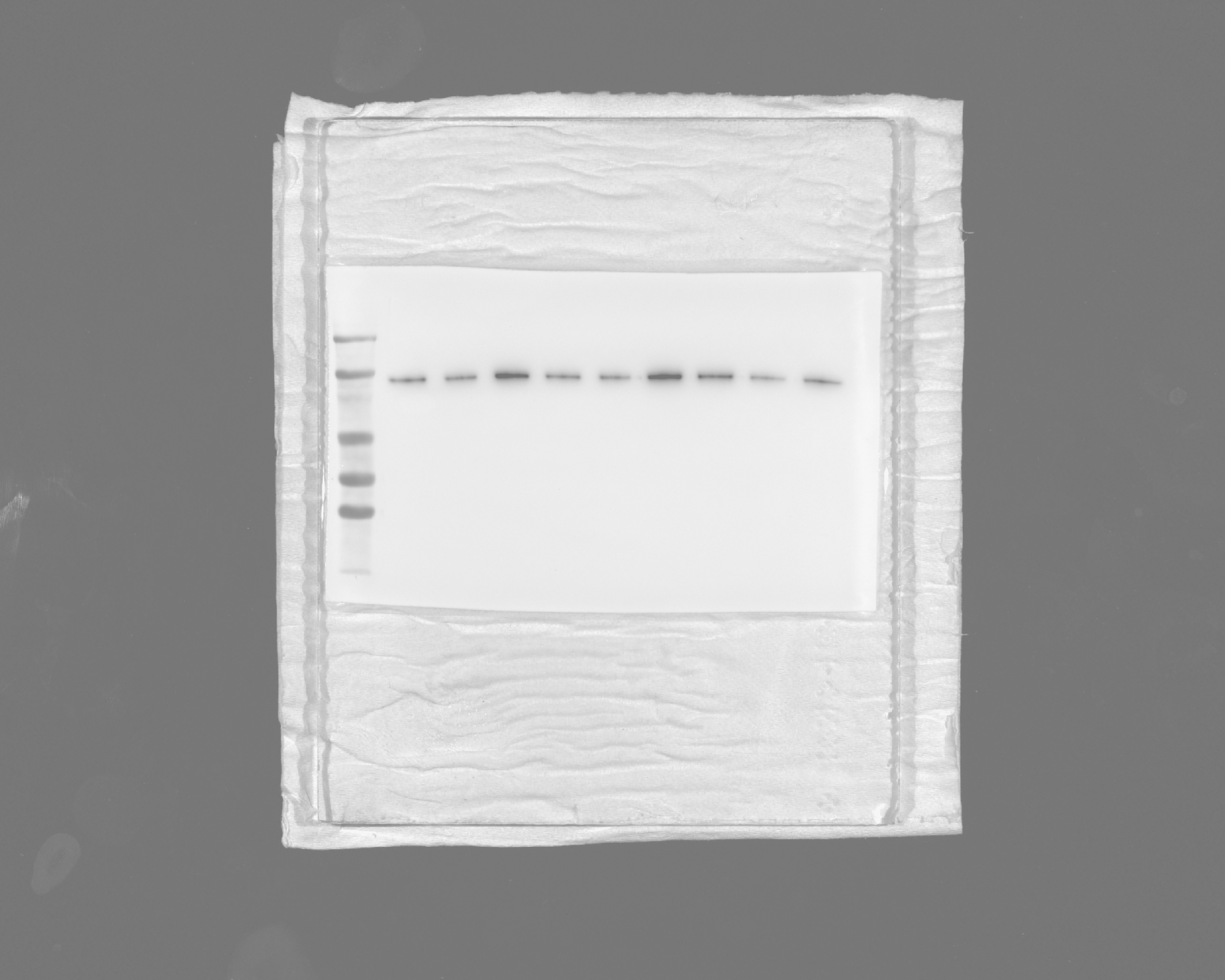

Supplement: Supplementary file 1 [file DataSheet_1.zip › Figure9/EPOR-original.tif]

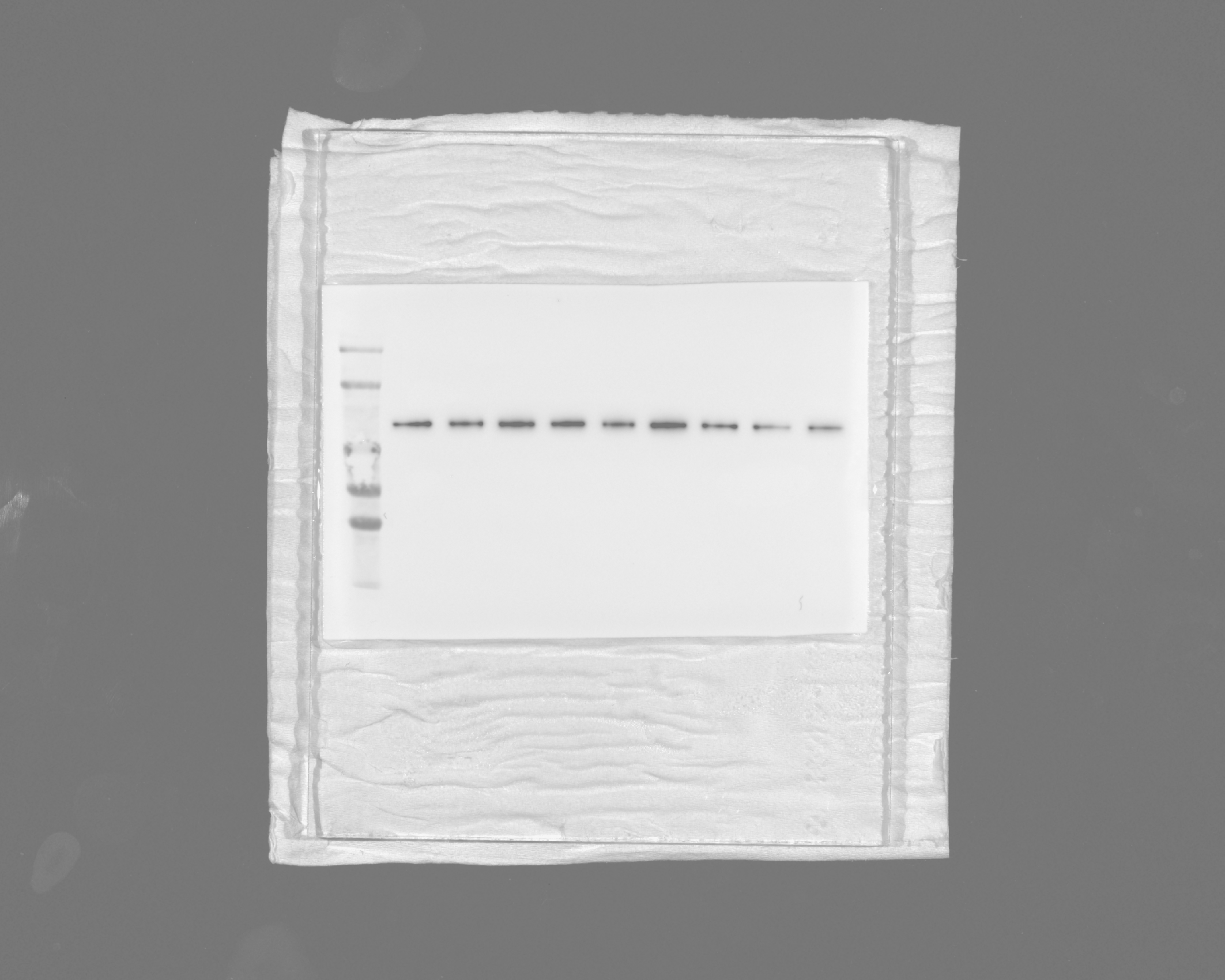

Supplement: Supplementary file 1 [file DataSheet_1.zip › Figure9/GATA1-original.tif]

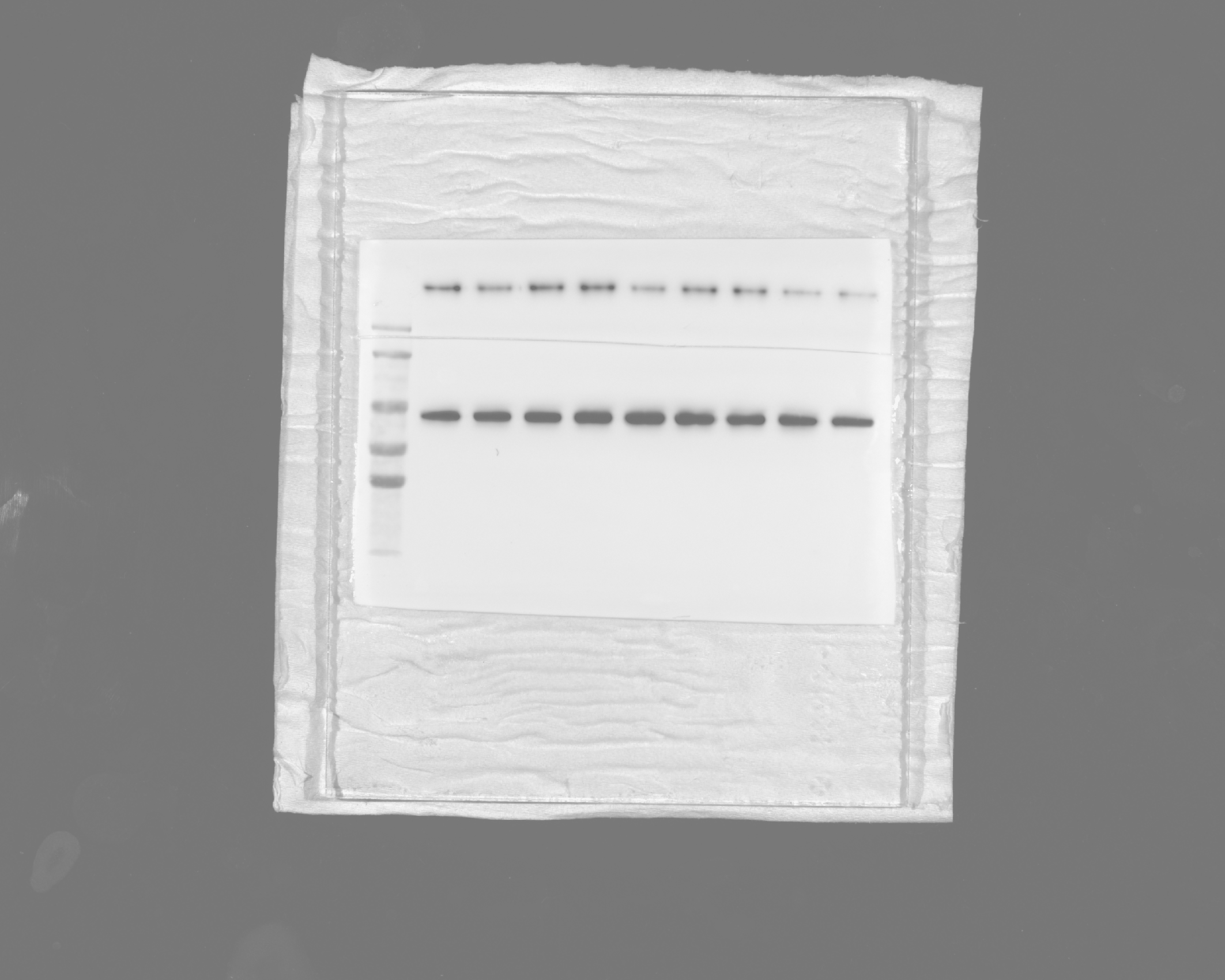

Supplement: Supplementary file 1 [file DataSheet_1.zip › Figure9/JAK1,GAPDH-original.tif]

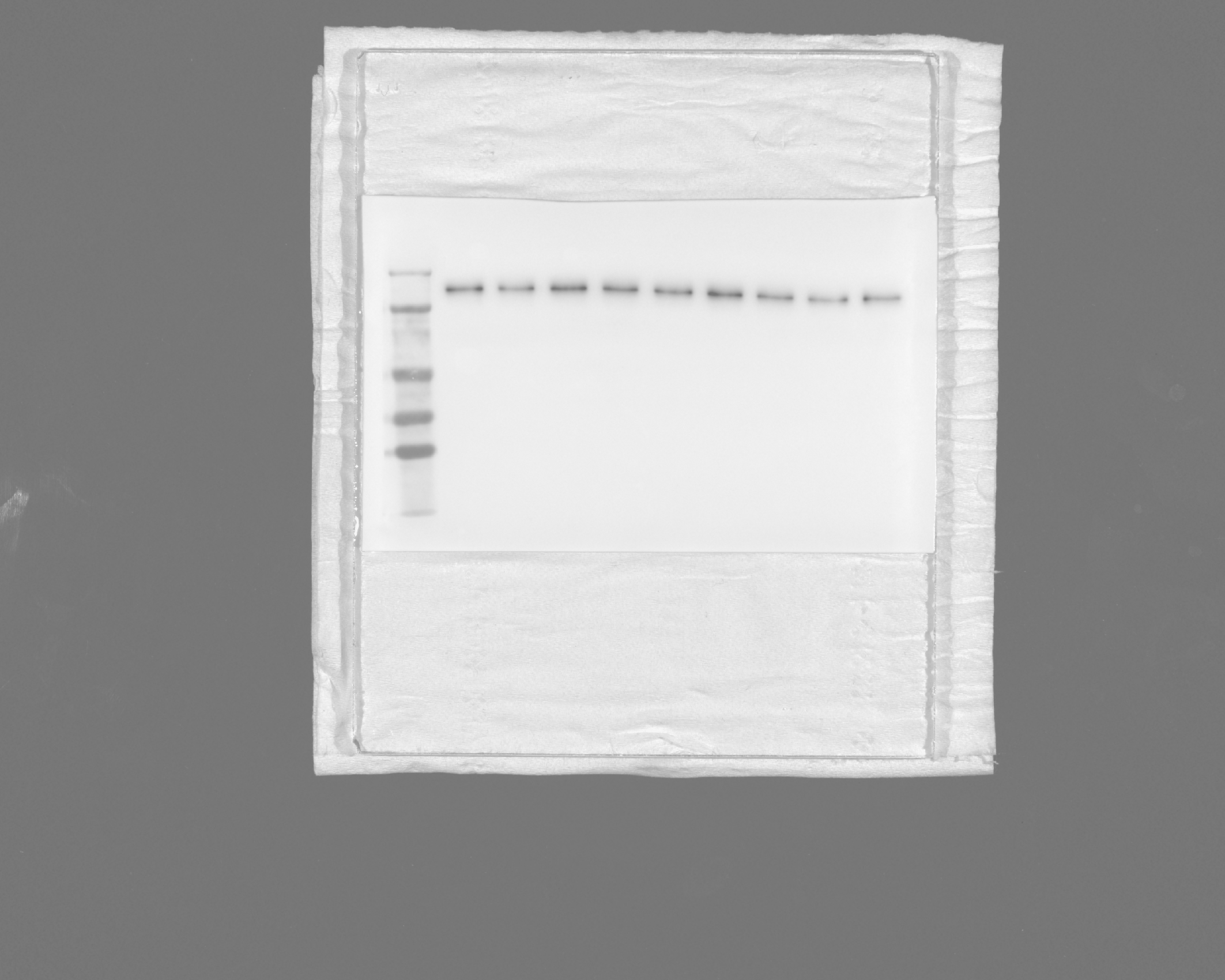

Supplement: Supplementary file 1 [file DataSheet_1.zip › Figure9/stat1-original.tif]
